# Combination effects of herbal and western medicines on osteoporosis in rheumatoid arthritis: systematic review and meta-analysis

**DOI:** 10.3389/fphar.2023.1164898

**Published:** 2023-08-10

**Authors:** Do Young Kwon, Ji Hyang Gu, Minseok Oh, Eun-Jung Lee

**Affiliations:** Department of Korean Rehabilitation Medicine, College of Korean Medicine, Daejeon University, Daejeon, Republic of Korea

**Keywords:** herbal medicine, meta-analysis, osteoporosis, rheumatoid arthritis, systematic review

## Abstract

**Purpose:** This study aimed to comprehensively review the effect of combining herbal medicine (HM) with Western Medicine (WM) compared to WM alone on bone mineral density (BMD) improvement for osteoporosis in patients with rheumatoid arthritis (RA).

**Methods:** Randomized controlled trials (RCTs) were searched using 10 databases, including PubMed, Embase, Cochrane Library, China National Knowledge Infrastructure, and Nation Information by NII. We selected studies that used BMD as an evaluation index and administered HM treatment for osteoporosis in patients with RA. Subsequently, a meta-analysis was conducted using BMD as a continuous variable using RevMan version 5.4.

**Results:** Eighteen RCTs that met the eligibility criteria of this study were selected. The total number of study participants was 1,491 (481 men and 1,010 women). The mean age of participants was 52.4 ± 7.4 years, and the mean morbidity period of RA was 6.8 ± 1.3 years. In all studies, disease-modifying anti-rheumatic drugs (DMARDs; 16 RCTs) or bisphosphonates (two RCTs) were used as WM co-intervention with HMs (17 types of HM, 18 RCTs). Overall, the combination of HM and WM improved the BMD score, producing better results than WM alone. In particular, when HM was used in combination with DMARDs, which were used in most studies, BMD improved by 0.04 g/cm^2^ (95% confidence interval [CI]: 0.03–0.05, *p* < 0.001, I^2^ = 19%) in the lumbar spine and 0.03 g/cm^2^ (95% CI: 0.02–0.03, *p* < 0.001, I^2^ = 0%) in the femoral neck compared to the DMARDs alone group after treatment. In addition to BMD, bone markers and inflammatory indicators evaluated by each RCT showed significant improvement after HM plus WM treatment. In the analysis of frequently prescribed HMs, the BMD after treatment was higher by 0.04 g/cm^2^ (95% CI: 0.03–0.04, *p* < 0.001, I^2^ = 45%) in the Xianlinggubao-capsule plus methotrexate (MTX) group and by 0.02 g/cm^2^ (95% CI: 0.00–0.03, *p* = 0.04, I^2^ = 0) in the Hanbikang-tang plus MTX group compared to the MTX alone group.

**Conclusion:** This systematic review cautiously provides evidence for the combined therapeutic effect of HM and WM for osteoporosis in patients with RA. However, well-designed, large-scale clinical trials are necessary before recommending this combination therapy for osteoporosis in patients with RA.

**Systematic Review Registration:** [https://www.crd.york.ac.uk/prospero/display_record.php?RecordID=331854], identifier [CRD42022331854].

## 1 Introduction

Osteoporosis is one of the most well-known extra-articular complications in patients with rheumatoid arthritis (RA). It increases the risk of fragility fractures, impairing the quality of life and life expectancy of patients with RA (Haugeberg et al., 2000a; Haugeberg et al., 2000b; Lodder et al., 2004; van Staa et al., 2006).

The global prevalence of osteoporosis in patients with RA was estimated to be 27.6% (95% confidence interval [CI]: 23.9–31.3) in 2022 (Moshayedi et al., 2022), approximately 1.5 times higher than that of the general population (19.7%; 95% CI: 18.0%–21.4%) (Xiao et al., 2022). Up to 50% higher prevalence of osteoporosis has been reported in postmenopausal women with RA compared to those without RA (Haugeberg et al., 2000a; Hauser et al., 2014). In particular, it has been observed that the risk of osteoporotic fracture in the femoral neck and spine is approximately twice as high in patients with RA than in the general population (Haugeberg et al., 2000a; Sinigaglia et al., 2000; Hoes et al., 2015; Choi et al., 2018). Moreover, patients with RA have longer fracture healing times and higher rates of complications, including non-unions, owing to systemic inflammatory conditions (Claes et al., 2012). Therefore, osteoporosis in patients with RA has become an important medical challenge to manage and prevent because it is a major disease that reduces the quality of life and increases the burden of medical expenses (Wu et al., 2021).

As the main risk factor for low bone mineral density (BMD) in patients with RA is a persistent inflammatory response, it is important to lower the inflammatory response to reduce disease activity and inhibit bone loss (Lodder et al., 2004; van Staa et al., 2006). Therefore, major societies for rheumatology recommend RA disease treatment as the primary treatment strategy for preventing bone loss in patients with RA (Singh et al., 2016; Smolen et al., 2017; Lau et al., 2019; Moshayedi et al., 2022). Treatments for bone loss in patients with RA, include disease-modifying anti-rheumatic drugs (DMARDs) such as methotrexate (MTX), leflunomide, and hydroxychloroquine as well as osteoporosis drugs, including bisphosphonates (BPs), and are used in clinics depending on the patient’s condition (Raterman et al., 2020). However, some RA standard treatments may cause osteoporosis as an adverse reaction (Machado-Alba et al., 2014; Lin et al., 2020) and have been reported to be ineffective in inhibiting bone loss (Mazzantini et al., 2000). Therefore, it is necessary to develop a safer and more effective treatment for inhibiting bone loss in patients with RA.

Herbal medicines (HMs) have been traditionally used for thousands of years and have been proven safe and accessible (Wang et al., 2021). Hence, they have been considered a complementary alternative treatment commonly used in patients with RA over the past decades (Huang et al., 2015; Daily et al., 2017; Li et al., 2019). In several recent studies, herbal extracts and their major compounds were found to act as anti-RA ingredients (Wang et al., 2021), and the combination of DMARDs and HMs significantly improved the disease activity of RA compared to DMARDs alone in patients with RA (Gong et al., 2021). In addition, some herbal extracts showed improvement in BMD and bone turnover markers via various mechanisms (He et al., 2017).

Based on the results of these previous studies, HMs may potentially be new therapeutic agents effective in preventing and treating osteoporosis in patients with RA. However, to our knowledge, no studies to date have analyzed the overall effect of HM on osteoporosis in patients with RA by reviewing previous studies. Therefore, a systematic review and a meta-analysis were conducted to address the research question of whether HMs are effective and safe in improving BMD in RA. Thus, this study suggested the possibility of HM as a new treatment for secondary osteoporosis.

## 2 Methods

This study was conducted according to the Preferred Reporting Items for Systematic Reviews and Meta-Analyses Statement. The review protocol was registered in the International Prospective Register of Systematic Reviews (Open Science Framework) with the Registration ID CRD42022331854.

### 2.1 Search strategy

Data from randomized controlled trials (RCTs) for patients with RA having osteoporosis published up to 24 April 2022, were searched and obtained without language restrictions. A total of 10 databases, including PubMed, Embase, Cochrane library, China National Knowledge Infrastructure, Wanfang, Nation Information by NII, KoreaMed, Kmbase, Korean studies Information Service System, and ScienceOn, were used to conduct a systematic review. The search keywords were (rheumat*[Title/Abstract] OR reumat*[Title/Abstract]), (osteoporo*[Title/Abstract] OR “bone loss” [Title/Abstract] OR “low bone densit*” [Title/Abstract] OR osteopeni*[Title/Abstract], and “Korean medicine*” [Title/Abstract] OR herb*[Title/Abstract] OR TCM [Title/Abstract] OR decoction*[Title/Abstract] OR “kampo medicine*” (Title/Abstract). These keywords were combined and modified as required, according to whether the database was in English, Chinese, and Korean. Detailed search strategies are reported in Supplementary Tables S1–S3.

### 2.2 Eligibility criteria

The inclusion criteria were as follows: 1) The study participants had RA with osteoporosis according to the criteria for osteoporosis defined by the World Health Organization in 1994 (WHO Study Group, 1994); 2) The intervention included HMs or herbal extracts taken orally with no restriction on the formulation; 3) The control group was administered WM (anti-osteoporosis or anti-rheumatic drugs) or placebo. There were no restrictions on the method of administration or whether supplements, such as calcium and vitamin D, were used; 4) Outcome measurement had to include the BMD value (lumbar spine or femoral neck) measured using dual-energy X-ray absorptiometry. There was no restriction on other frequently used measurements in the included studies; 5) The study design had to be an RCT on humans. The following studies were excluded: 1) Studies without full texts or those where the BMD measurement site was not specified; 2) Studies where BMD could not be estimated using dual-energy X-ray absorptiometry; 3) Studies with a Jadad score <2 points, which was judged to indicate low quality. Additionally, unpublished studies and ongoing trials were not considered in this study.

### 2.3 Review process and data extraction

Two independent reviewers (DK and JG) checked the title and abstract of the searched articles and primarily screened them according to the inclusion and exclusion criteria. After obtaining the full text of RCTs, the full text of the first selected studies was rechecked and the studies to be analyzed were finally determined. In cases of disagreement between the two reviewers, the corresponding studies were reviewed together until agreement was reached. In cases where agreement was not reached, the opinion of a third reviewer (E-JL) was adopted. The reviewers extracted the characteristics of RCTs (characteristics of participants, such as sex and age; sample size; and duration of RA), interventions (type of HM, composition of prescription, dose, extraction process, quality control, chemical analysis, and treatment duration), and control (BMD score, bone turnover markers, inflammatory indicators, and adverse events), and performed a quality assessment of studies. When extracting data of the botanical drugs used in each HM treatment, the notation of botanic drugs was as shown in “*scientific plant name”* [family; synonyms]. In cases of animal-derived material, the scientific name and the used part were presented together. In addition, an analysis was performed with respect to the frequency of HMs used in RCTs.

### 2.4 Statistical analysis

All mean data described in this study regarding the characteristics of participants and interventions are the mean of means, which were presented as the mean and standard deviation (SD) computed by Microsoft Excel software.

### 2.5 Meta-analysis

In this study, a meta-analysis was conducted using BMD values as the primary outcome to draw meaningful conclusions regarding the clinical effect of HMs on BMD. These analyses were conducted in terms of comparison between the HM with WM group and the WM alone group before and after administration of HMs and WMs (before and after BMD). First, a meta-analysis was performed on the BMD data of the lumbar spine and femoral neck. In the analysis of the HM plus WM group and WM alone group, the BMDs of the lumbar spine and femoral neck showed high heterogeneity. Therefore, the included RCTs were classified based on the use of DMARDs and BPs and according to the type of drugs administered in the treatment and control groups. Subsequently, a meta-analysis was conducted to compare the final value of BMD after the intervention. We conducted a meta-analysis to confirm the effect of the prescriptions frequently used in the selected studies on the difference in BMD.

However, when subgroup analysis was performed on BMD of the lumbar spine and femoral neck of studies classified according to the type of WM used, the heterogeneity of the merged results was very high. Therefore, we conducted a meta-analysis for each type of WM and each BMD measurement site. Nevertheless, due to the high heterogeneity (Supplementary Figures S1, S2), additional sensitivity analysis was conducted, and as a result, some studies (Wang, 2018a; Wang and Liu, 2018b; Liu, 2018) were found to be the cause of heterogeneity. For the reliability of the results, we conducted a meta-analysis excluding three studies that caused heterogeneity.

In addition, for a comprehensive evaluation of the improvement of inflammatory indicators, a meta-analysis was conducted using inflammatory indicators (erythrocyte sedimentation rate [ESR] and C-reactive protein [CRP] as secondary outcomes). The validity rate, an outcome used in most selected studies, was excluded from the meta-analysis because there were differences in the standards and calculation methods used in each study.

The analysis program used was Cochrane’s Review Manager (RevMan) 5.4 (The Nordic Cochrane Centre, The Cochrane Collaboration, Copenhagen, Denmark). The BMD scores, ESR, and CRP were considered continuous variables, and a meta-analysis was conducted with a 95% CI using the SD and mean of the final value. A fixed-effect (I^2^<50%) or a random-effect model (I^2^ ≥ 50%) was applied according to the I^2^ value obtained by Higgin’s homogeneity test.

### 2.6 Quality assessment

Two reviewers (DK and JG) assessed the quality of studies by a Jadad score evaluating randomization, blinding, withdrawals, and dropouts of study participants (Jadad et al., 1996). In the final selected studies, Cochrane’s Risk of bias 2 (RoB 2) (Cochrane Handbook for Systematic Reviews of Interventions 6.3) was used to assess the risk of bias (Higgins and Green, 2011).

To assess the quality of evidence of each meta-analysis result, we used the Grading of Recommendations, Assessment, Development, and Evaluations approach (Guyatt et al., 2008).

## 3 Results

### 3.1 Characteristics of the included RCTs

**FIGURE 1:**
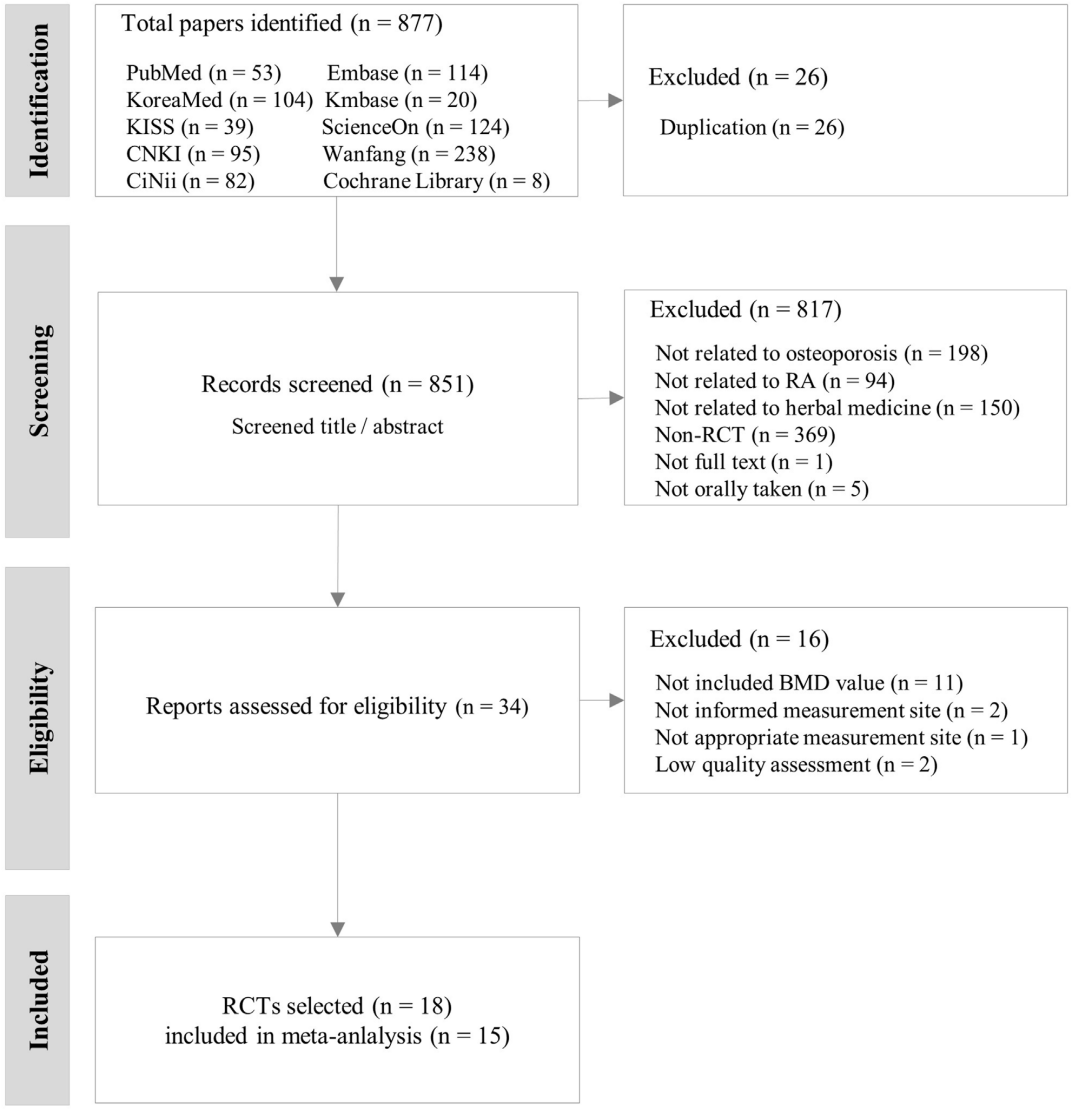
Flow chart of the study selection process. BMD: bone mineral density, RA: rheumatoid arthritis, RCT: randomized controlled trial.

Of the 851 RCTs, 18 were selected according to the eligibility criteria (Figure 1). All selected studies had been published after 2015. In the 18 RCTs, the total number of participants was 1,491 (481 men and 1,010 women), with 731 in the intervention group and 760 in the control group. The average number of participants was 82.8 ± 22.6 (an average of 26.1 ± 11.7 for men and 56.1 ± 18.7 for women). The average mean age of participants presented in each study was 52.4 ± 7.4 years, and the mean RA duration was 6.8 ± 1.3 years (Table 1).

**TABLE 1:** Summary of the included RCT data.

Author (years)	N. Of participants (I/C)	Mean age (years)	Duration of RA (years)	Intervention* (HM + control)	Control*	Period (Mon)	Initial BMD (g/cm^2^)	Last BMD (g/cm^2^)	BMD and RA-related outcome	Jadad score
	Male/Female	(I/C)	(I/C)				(I/C)	(I/C)		
***Herbal Medicines + DMARDs (16 RCTs)***										
Liu (2015)	60 (30/30) 21/39	NR	7.6 ± 2.4(NR/NR)	Gulong-capsule 6,000 mg/d + C	MTX (7,500–10000) SSZ (15,750)	12	L: 0.610 ± 0.110/0.630 ± 0.080 F: 0.590 ± 0.060/0.600 ± 0.090	L: 0.750 ± 0.140/0.690 ± 0.110^ab^ F: 0.730 ± 0.130/0.670 ± 0.120^ab^	(1)^ab^ (2)^ab^ (3)^ab^ (4)^ab^	2
Pang et al. (2015)	112 (56/56) 28/84	43.8 ± 5.6/43.5 ± 5.3	7.4 ± 2.5/7.6 ± 2.4	Hanbikang-tang 250 mL/d + C	MTX (10), HCQ (2,800)	6	L: 0.776 ± 0.120/0.774 ± 0.110 F: 0.715 ± 0.080/0.718 ± 0.080	L: 0.812 ± 0.110/0.789 ± 0.120^b^ F: 0.742 ± 0.110/0.731 ± 0.090^b^	(1)^ab^ (2)^ab^ (3)^ab^ (4)^a^	2
Xie and Wang, (2017)	86 (43/43) 41/45	45.2 ± 6.2/45.5 ± 6.4	6.3 ± 1.6/6.1 ± 1.4	Decoction 200 mL/d + C	MTX (10), HCQ (2,800)	3	NR	L: 0.813 ± 0.120/0.756 ± 0.100^b^	(1)^b^ (2)^b^ (3)^b^ (4)^b^	3
Shu (2017)	72 (37/35) 12/60	48.2 ± 9.3/46.8 ± 11.5	5.4 ± 2.1/4.7 ± 1.4	Hanbikang-tang 2 pack/d + LEF (140) MTX (10) (Diclofenac sodium 75 mg/d prn)	LEF (140), MTX (10), Calcium (7,500) Vit D3 875 IU/w Alfacalcidol (0.0035) (Diclofenac sodium 75 mg/d prn)	3	L: 0.829 ± 0.082/0.821 ± 0.077 F: 0.693 ± 0.084/0.706 ± 0.114	L: 0.837 ± 0.042/0.826 ± 0.063 F: 0.714 ± 0.072/0.698 ± 0.094	(1)^ab^ (2)^ab^ (3)^ab^ (5)^ab^	3
Liu (2018)	75 (37/38) 13/62	48.5 ± 9.3/49.1 ± 10.3	5.9 ± 2.5/5.7 ± 2.4	Buxuerongjin-tang 250 mL/d + C	LEF (140) Calcium (10,500) Vit D3 875 IU/w Alfacalcidol (0.0035) (Diclofenac sodium 75 mg/d prn)	6	L: 0.973 ± 0.792/0.959 ± 0.549 F: 0.863 ± 0.045/0.839 ± 0.049	L: 1.061 ± 0.087/0.988 ± 0.062^ab^ F: 0.931 ± 0.052/0.847 ± 0.030^ab^	(1)^ab^ (2)^ab^ (3)^ab^ (4)^ab^ (5)^ab^	3
Luo et al. (2018)	48 (24/24) 25/23	51.1 ± 10.8/49.5 ± 10.0	8.4 ± 3.1/9.7 ± 4.1	Xianlinggubao-capsule 3,000 mg/d + C	MTX (10), LEF (70) Calcium (21,000) Vit D3 1750 IU/w, Alfacalcidol (0.00175)	2	L: 0.727 ± 0.186/0.715 ± 0.198 F: 0.808 ± 0.200/0.794 ± 0.164	L: 0.992 ± 0.170/0.852 ± 0.183^ab^ F: 0.996 ± 0.155/0.987 ± 0.112^a^	None	2
Pang et al. (2018)	120 (60/60) 31/89	44.7 ± 4.7/44.6 ± 5.7	7.6 ± 2.3/7.6 ± 2.4	Guweian-tang 400 mL/d + C	MTX (15), LEF (140), Folate (70)	6	L: 0.77 ± 0.13/0.78 ± 0.12 F: 0.72 ± 0.08/0.72 ± 0.07	L: 0.820 ± 0.120/0.790 ± 0.130^ab^ F: 0.750 ± 0.110/0.730 ± 0.080^ab^	None	3
Tian et al. (2018b)	80 (40/40) 12/68	56.4 ± 8.9/55.8 ± 9.2	6.8 ± 2.1/6.7 ± 1.9	Guizhifuzi-tang 400 mL/d + C	Yisaipu (50) Alfacalcidol (0.0035) Calcium (5,250) Vit D 700 IU/w	3	L: 0.987 ± 0.252/0.979 ± 0.315 F: 0.846 ± 0.026/0.851 ± 0.028	L: 1.120 ± 0.314/0.993 ± 0.353^ab^ F: 0.897 ± 0.031/0.872 ± 0.023^ab^	(5)^ab^	2
Tian et al. (2018b)	80 (40/40) 8/72	61.4 ± 7.0/59.7 ± 7.2	8.8 ± 3.9/8.4 ± 2.9	Gulaoyukang-pills 3,600 mg/d + C	MTX (10) Calcium (10,500) Vit. D 43,400 IU/w	6	L: 0.720 ± 0.060/0.720 ± 0.090	L: 0.810 ± 0.080/0.750 ± 0.090^ab^	(1)^a^ (2)^a^ (3)^a^ (4)^a^	3
Wang, (2018a)	108 (54/54) 39/69	48.6 ± 6.5/48.3 ± 5.0	7.7 ± 2.3/7.5 ± 2.2	Bushentongluo-recipe 250 mL/d + C	MTX (35–70) LEF initial 1–3 days 50 mg/d, after that 10–20 mg/d	2	L: 0.750 ± 0.140/0.760 ± 0.140 F: 0.770 ± 0.120/0.760 ± 0.190	L: 0.820 ± 0.020/0.770 ± 0.020^ab^ F: 0.810 ± 0.020/0.730 ± 0.020^ab^	(1)^ab^ (2)^ab^ (3)^ab^ (4)^ab^	3
Wang and liu, (2018b)	126 (63/63) 54/72	54.9 ± 5.3/55.2 ± 2.1	5.4 ± 0.9/5.2 ± 1.0	Bushenkangfengshi-recipe 250 mL/d + C	MTX (10)	6	NR	L: 0.789 ± 0.110/0.681 ± 0.100^b^	(1)^b^ (2)^b^ (3)^b^	2
Jiang et al. (2019)	60 (30/30) 23/37	54 ± 6/55 ± 6	6.4 ± 2.4/6.8 ± 2.7	Biqufeng-drink 300 mL/d + C	MTX (10)	3	L: 0.620 ± 0.070/0.620 ± 0.090 F: 0.600 ± 0.080/0.600 ± 0.090	L: 0.750 ± 0.100/0.700 ± 0.080^ab^ F: 0.740 ± 0.110/0.680 ± 0.100^ab^	(1)^ab^ (2)^ab^ (3)^ab^ (4)^ab^	3
Zhao and Xu, (2019)	96 (48/48) 37/59	42.6 ± 15.4/43.4 ± 15.7	8.5 ± 2.9/8.4 ± 2.8	Qianggu-capsule 750 mg/d + C	MTX (35–70) SSZ starting dose 2000–3,000 mg/d, maintenance dose 1,500–2000 mg/d Calcium (10,500–21000) Vit D3 875–1750 IU/w	3	L: 0.770 ± 0.110/0.750 ± 0.100 F: 0.730 ± 0.090/0.740 ± 0.100	L: 0.850 ± 0.130/0.790 ± 0.110^ab^ F: 0.740 ± 0.120/0.750 ± 0.090	(1)^ab^ (2)^ab^ (3)^ab^ (4)^ab^	2
Zhou (2019)	90 (45/45) 39/51	57.1 ± 4.0/56.3 ± 4.2	6.5 ± 1.4/6.2 ± 1.5	Xianlinggubao-capsule 3,000 mg/d + C	MTX (5–20)	2	NR	L: 0.815 ± 0.023/0.774 ± 0.018^b^ F: 0.762 ± 0.022/0.733 ± 0.020^b^	(1)^b^ (2)^b^ (3)^b^ (4)^b^	3
Zhu and He, (2019)	58 (29/29) 21/37	62.0 ± 5.8/61.2 ± 5.7	4.9 ± 0.8/4.1 ± 0.5	Xianlinggubao-capsule 3,000 mg/d + C	MTX (5–20)	2	L: 0.718 ± 0.100/0.717 ± 0.110 F: 0.770 ± 0.130/0.762 ± 0.120	L: 0.750 ± 0.160/0.742 ± 0.150^ab^ F: 0.814 ± 0.140/0.798 ± 0.130^ab^	(1)^ab^ (3)^ab^ (4)^ab^	2
Quan et al. (2020)	80 (40/40) 23/57	54.7 ± 6.0/53.2 ± 5.1	6.7 ± 1.6/6.4 ± 1.1	Hanbikang-tang 1 pack/d + C	MTX (15) Calcium (21,000) Vit D3 1750 IU/w Alfacalcidol (0.0035) Diclofenac sodium (700)	3	L: 0.768 ± 0.110/0.766 ± 0.090 F: 0.712 ± 0.070/0.713 ± 0.080	L: 0.816 ± 0.120/0.785 ± 0.130^ab^ F: 0.750 ± 0.090/0.734 ± 0.090^ab^	(1)^ab^ (2)^ab^ (3)^ab^ (4)^ab^ (5)^ab^	3
***Herbal Medicines + BPs (Two RCTs)***										
Qiu et al. (2017)	90 (30/30/30) 25/65	71.4 ± 3.5/72.3 ± 4.2	NR	Jingu-capsule 9 pills/d + C	Pamidronate disodium 90 mg/5% DW 750 mL/m Calcium (10,500) Vit D3 875 IU/w Calcitriol (0.00175)	6	L: 0.581 ± 0.056/0.562 ± 0.042 F: 0.596 ± 0.047/0.546 ± 0.022	L: 0.770 ± 0.044/0.633 ± 0.053^ab^ F: 0.862 ± 0.032/0.626 ± 0.082^ab^	(1)^ab^	2
Wang (2019)	50 (25/25) 29/21	58.5 ± 3.4/58.6 ± 3.5	6.6 ± 1.3/6.9 ± 1.2	Gushukang, Xianlinggubao-capsule, Jintiange bid + C	Alendronate sodium Ibandronate sodium	2	L: 0.710 ± 0.030/0.720 ± 0.080 F: 0.630 ± 0.040/0.640 ± 0.050	L: 0.890 ± 0.070/0.760 ± 0.020^ab^ F: 0.850 ± 0.040/0.740 ± 0.050^ab^	None	2
Average	82.8 ± 22.6 26.1 ± 11.7/56.1 ± 18.7	52.4 ± 7.4	6.8 ± 1.3			4.2 ± 2.5	L: 0.766 ± 0.104/0.752 ± 0.108 F: 0.717 ± 0.086/0.714 ± 0.087	L: 0.848 ± 0.102/0.782 ± 0.090 F: 0.806 ± 0.082/0.755 ± 0.087		

5% DW: 5% dextrose in water solution, BMD: bone mineral density, BP: bisphosphonate, C: control group, Calcium: calcium carbonate, d: day, DMARDs: disease-modifying anti-rheumatic drugs, F: femoral neck BMD, HCQ: hydroxychloroquine sulfate, HM: herbal medicine, I: intervention group, L: lumbar spine BMD, LEF: leflunomide, MTX: methotrexate, N: number, NR: not reported, mon: month, RA: rheumatoid arthritis, RCT: randomized controlled trial, SSZ: sulfasalazine, w: week.

*If the unit was mg/w, it was marked as ( ), and other cases were specified as it was.

^a^ *p*<0.05 compared to pre-treatment.

^b^ *p*<0.05 compared to the control group after treatment, (1): pain scale (included one of the joint tenderness index, painful joint count, visual analogue scale [VAS]), (2): swelling scale (included one of the joint swelling index or swollen joint count), (3): morning stiffness scale (used one of min or hours), (4): function scale (included one of the joint function index, degree of weakness of the limb, average grip strength of both hands, or 15-m walking time (s)), (5): disease activity score with 28-joint assessment (DAS28).

All intervention groups received combined treatment with HM and WM, whereas the control groups received WM alone. The prescribed WM was classified into 16 DMARDs (seven RCTs with DMARDs and nine RCTs with DMARDs plus supplements), and two bisphosphonates (BPs) (one RCT with alendronate sodium, ibandronate sodium and one RCT with pamidronate disodium plus supplements). The average duration of treatment was 4.2 ± 2.5 months (Table 1).

The mean initial BMD value of 15 studies, excluding three studies without initial BMD records, was 0.766 ± 0.104 g/cm^2^ in the lumbar spine; 0.717 ± 0.086 g/cm^2^ in the femoral neck in the experimental group and 0.752 ± 0.708 g/cm^2^ in the lumbar spine; 0.714 ± 0.087 g/cm^2^ in the femoral neck in the control group. No significant difference was observed between the groups in each study (*p* > 0.05) (Table 1).

All RCTs used BMD as an outcome measurement; therefore, we set BMD as the primary outcome. In addition to BMD, bone markers and inflammatory indicators were used as evaluation indicators in the selected RCTs. Bone markers included one bone resorption marker (C-telopeptide of type 1 collagen [CTX-I] in three studies), three bone formation markers (alkaline phosphatase [ALP] in three studies, bone alkaline phosphatase [BALP] in eight studies, and bone gamma-carboxyglutamic acid protein [BGP] in four studies), five mineral composition and metabolic markers (calcium in nine studies, phosphorus in four studies, 25-hydroxyvitamin D [25-OHD] in two studies, urinary calcium/creatinine [Ca/Cr] in three studies, and parathyroid hormone [PTH] in three studies), and three cytokines (receptor activator of nuclear factor κB ligand [RANKL] in one study, osteoprotegerin [OPG] in two studies, and RANKL/OPG in one study). As inflammatory indicators, anti-cyclic citrullinated peptide [anti-CCP], rheumatoid factor [RF], ESR, and CRP were used in three, four, eight, and five studies, respectively (Figure 8).

### 3.2 Analysis of HMs used in the RCTs

A total of 17 types of HMs were used in the 18 RCTs, all of which were multi-HMs. The formulations of the 17 HM were capsules in four, decoctions in nine, pill in one, and unknown in three RCTs. Except for one RCT that selected three HMs, other RCTs applied one treatment (one HM). Six studies used the same prescription name and composition: Hanbikang-tang (HBK) in three studies (Pang et al., 2015; Shu, 2017; Quan et al., 2020) and Xianlinggubao-capsule (XLGB) in three studies. Of the three studies that used HBK, two (Shu, 2017; Quan et al., 2020) used the same drug dose but one (Pang et al., 2015) used a different dose. The XLGB used in three studies (Luo et al., 2018; Zhou, 2019; Zhu and He, 2019) had the same China Food and Drug Administration registration number; therefore, they were considered the same prescription. However, the dose of each botanical drug was unknown (Table 2).

**TABLE 2:** The composition of botanical drugs in each HM.

Name of HM (type of formula, N. of botanical drugs)	Source	Extraction process	Composition of HMs (g/day)	Quality control report	Chemical analysis report
*Herbal Medicines + DMARDs*					
Gulong capsule (GL, Capsule, NR)	Shandong Dong-e E-Jiao pharmaceutical	NR	NR	NR	NR
Hanbikang decoction* (HBK, Decoction, 9)	Ruikang Hospital Affiliated with Guangxi University of Chinese Medicine	Partially reported^b^	*Gentiana macrophylla* Pall. [Gentianaceae; Gentianae Macrophyllae R.] (15), *Astragalus mongholicus* Bunge [Fabaceae; Astragali R.] (15), *Sinomenium acutum* (Thunb.) Rehder & E.H.Wilson [Menispermaceae; Sinomenii Caulis et Rh.] (15), *Saposhnikovia divaricata* (Turcz. ex Ledeb.) Schischk. [Apiaceae; Saposhnikoviae R.] (10), *Aconitum carmichaelii* Debeaux [Ranunculaceae; Aconiti Lateralis R. Preparata] (10), *Ephedra sinica* Stapf [Ephedraceae; Ephedrae H.] (10), *Angelica sinensis* (Oliv.) Diels [Apiaceae; Angelica sinensis R.] (10), *Epimedium brevicornu* Maxim. [Berberidaceae; Epimedii H.] (20), *Cibotium barometz* (L.) J.Sm. [Cyatheaceae; Cibotii Rh.] (20)	NR	NR
Unnamed HM (Decoction, 9)	Yangchun Hospital of TCM	Partially reported^b^	*Cibotium barometz* (L.) J.Sm. [Cyatheaceae; Cibotii Rh.] (20), *Epimedium brevicornu* Maxim. [Berberidaceae; Epimedii H.] (20), *Astragalus mongholicus* Bunge [Fabaceae; Astragali R.] (15), *Gentiana macrophylla* Pall. [Gentianaceae; Gentianae Macrophyllae R.] (15), *Sinomenium acutum* (Thunb.) Rehder & E.H.Wilson [Menispermaceae; Sinomenii Caulis et Rh.] (15), *Aconitum carmichaelii* Debeaux [Ranunculaceae; Aconiti Lateralis R. Preparata] (10), *Angelica sinensis* (Oliv.) Diels [Apiaceae; Angelica sinensis R.] (10), *Ephedra sinica* Stapf [Ephedraceae; Ephedrae H.] (10)	NR	NR
Hanbikang decoction* (HBK, Decoction, 9)	Peili (Nanning) pharmaceutical (Shu, 2017) Guangdong Yifang pharmaceutical (Quan et al., 2020)	Partially reported^c^	*Gentiana macrophylla* Pall. [Gentianaceae; Gentianae Macrophyllae R.] (10), *Sinomenium acutum* (Thunb.) Rehder & E.H.Wilson [Menispermaceae; Sinomenii Caulis et Rh.] (15), *Saposhnikovia divaricata* (Turcz. ex Ledeb.) Schischk. [Apiaceae; Saposhnikoviae R.] (10), *Aconitum carmichaelii* Debeaux [Ranunculaceae; Aconiti Lateralis R. Preparata] (10), *Epimedium brevicornu* Maxim. [Berberidaceae; Epimedii H.] (15), *Cibotium barometz* (L.) J.Sm. [Cyatheaceae; Cibotii Rh.] (10), *Astragalus mongholicus* Bunge [Fabaceae; Astragali R.] (10), *Ephedra sinica* Stapf [Ephedraceae; Ephedrae H.] (10), *Angelica sinensis* (Oliv.) Diels [Apiaceae; Angelica sinensis R.] (10)	NR	NR
Buxuerongjin decoction (BXRJ, Decoction, 13)	Ruikang Hospital Affiliated with Guangxi University of Chinese Medicine	Partially reported^b^	*Rehmannia glutinosa* (Gaertn.) DC. [Orobanchaceae; Rehmanniae R. Preparata] (10), *Cistanche deserticola* Ma [Orobanchaceae; Cistanchis H.] (15), *Gentiana macrophylla* Pall. [Gentianaceae; Gentianae Macrophyllae R.] (10), *Cuscuta chinensis* Lam. [Convolvulaceae; Cuscutae S.] (15), *Achyranthes bidentata* Blume [Amaranthaceae; Achyranthis R.] (12), *Eucommia ulmoides* Oliv. [Eucommiaceae; Eucommiae C.] (10), *Taxillus chinensis* (DC.) Danser [Loranthaceae; Taxilli Ra.] (15), *Cibotium barometz* (L.) J.Sm. [Cyatheaceae; Cibotii Rh.] (8), *Chaenomeles speciosa* (Sweet) Nakai [Rosaceae; Chaenomelis F.] (10), *Angelica biserrata* (R.H.Shan & C.Q.Yuan) C.Q.Yuan & R.H.Shan [Apiaceae; Angelicae Pubescentis R.] (15), *Saposhnikovia divaricata* (Turcz. ex Ledeb.) Schischk. [Apiaceae; Saposhnikoviae R.] (10), *Panax ginseng* C.A.Mey. [Araliaceae; Ginseng R.] (10), *Angelica sinensis* (Oliv.) Diels [Apiaceae; Angelica sinensis R.] (15)	NR	NR
Xianlinggubao capsule (XLGB, Capsule, 6)	Sinopharm Group Tongjitang (Guizhou) pharmaceutical	NR	*Epimedium brevicornu* Maxim. [Berberidaceae; Epimedii H.], *Dipsacus asper* Wall. ex DC. [Caprifoliaceae; Dipsaci R.], *Salvia miltiorrhiza* Bunge [Lamiaceae; Salviae Miltiorrhizae R.], *Anemarrhena asphodeloides* Bunge [Asparagaceae; Anemarrhenae Rh.], *Cullen corylifolium* (L.) Medik. [Fabaceae; Psoraleae S.], *Rehmannia glutinosa* (Gaertn.) DC. [Orobanchaceae; Rehmanniae R.] (NR)	NR	NR
Guweian-tang (GWA, Decoction, 18)	Ruikang Hospital Affiliated with Guangxi University of Chinese Medicine	Partially reported^c^	*Astragalus mongholicus* Bunge [Fabaceae; Astragali R.], *Poria cocos* Wolf [Polyporaceae; Poria Sclerotium], *Dioscorea oppositifolia* L. [Dioscoreaceae; Dioscoreae Rh.], *Cuscuta chinensis* Lam. [Convolvulaceae; Cuscutae S.], *Polygonatum sibiricum* Redouté [Asparagaceae; Polygonati Rh.], *Morus alba* L. [Moraceae; Mori F.], Hornes of *Cervus nippon* Temminck [Cervidae; Cervi Cornus Colla], Placenta of *Homo sapiens* L. [Hominidae; Hominis Placenta], *Epimedium brevicornu* Maxim. [Berberidaceae; Epimedii H.], *Cibotium barometz* (L.) J.Sm. [Cyatheaceae; Cibotii Rh.], *Cistanche deserticola* Ma [Orobanchaceae; Cistanchis H.], *Cullen corylifolium* (L.) Medik. [Fabaceae; Psoraleae S.], *Achyranthes bidentata* Blume [Amaranthaceae; Achyranthis R.], *Eucommia ulmoides* Oliv. [Eucommiaceae; Eucommiae C.], *Spatholobus suberectus* Dunn [Fabaceae; Spatholobi Caulis]*, Carthamus tinctorius* L. [Asteraceae; Carthami Fl.] (NR)	NR	NR
Guizhifuzi decoction (GZFZ, Decoction, 11)	Baoji Central Hospital	Partially reported^a^	*Neolitsea cassia* (L.) Kosterm. [Lauraceae; Cinnamomi Ra.] (20), *Aconitum carmichaelii* Debeaux [Ranunculaceae; Aconiti Lateralis R. Preparata] (30), *Paeonia lactiflora* Pall. [Paeoniaceae; Paeoniae R. Rubra] (15), *Ziziphus jujuba* Mill. [Rhamnaceae; Zizyphi F.] (15), *Impatiens balsamina* L. [Balsaminaceae; Impatientis S.] (15), *Achyranthes bidentata* Blume [Amaranthaceae; Achyranthis R.] (20), Dried body of *Zaocys dhumnades* Cantor [Colubridae; Zaocys] (20), *Boswellia sacra* Flück. [Burseraceae; Olibanum] (15), *Commiphora myrrha* (T.Nees) Engl. [Burseraceae; Myrrha] (15), *Angelica sinensis* (Oliv.) Diels [Apiaceae; Angelica sinensis R.] (20), *Spatholobus suberectus* Dunn [Fabaceae; Spatholobi Caulis] (30)	NR	NR
Gulaoyukang pills (GLYKP, pill, NR)	Gansu Food & Drug Administration	NR	*NR*	NR	NR
Bushentongluo recipe (BSTL, Decoction, 11)	Yingxian Hospital of TCM	Partially reported^b^	*Epimedium brevicornu* Maxim. [Berberidaceae; Epimedii H.] (20), *Cullen corylifolium* (L.) Medik. [Fabaceae; Psoraleae S.] (15), *Drynaria roosii* Nakaike [Polypodiaceae; Drynariae Rh.] (15), *Rehmannia glutinosa* (Gaertn.) DC. [Orobanchaceae; Rehmanniae R. Preparata] (12), *Cornus officinalis* Siebold & Zucc. [Cornaceae; Corni F.] (12), *Salvia miltiorrhiza* Bunge [Lamiaceae; Salviae Miltiorrhizae R.] (10), *Eucommia ulmoides* Oliv. [Eucommiaceae; Eucommiae C.] (10), *Spatholobus suberectus* Dunn [Fabaceae; Spatholobi Caulis] (10), *Dipsacus asper* Wall. ex DC. [Caprifoliaceae; Dipsaci R.] (10), *Angelica sinensis* (Oliv.) Diels [Apiaceae; Angelica sinensis R.] (10), *Glycyrrhiza uralensis* Fisch. ex DC. [Fabaceae; Glycyrrhizae R. et Rh.] (5)	NR	NR
Bushenkangfengshi recipe (BSKFS, Decoction, 9)	Jiaozuo No.2 People’s Hospital	Partially reported^a^	*Epimedium brevicornu* Maxim. [Berberidaceae; Epimedii H.] (20), *Cibotium barometz* (L.) J.Sm. [Cyatheaceae; Cibotii Rh.] (20), *Gentiana macrophylla* Pall. [Gentianaceae; Gentianae Macrophyllae R.] (15), *Astragalus mongholicus* Bunge [Fabaceae; Astragali R.] (15), *Sinomenium acutum* (Thunb.) Rehder & E.H.Wilson [Menispermaceae; Sinomenii Caulis et Rh.] (15), *Saposhnikovia divaricata* (Turcz. ex Ledeb.) Schischk. [Apiaceae; Saposhnikoviae R.] (10), *Aconitum carmichaelii* Debeaux [Ranunculaceae; Aconiti Lateralis R. Preparata] (10), *Ephedra sinica* Stapf [Ephedraceae; Ephedrae H.] (10), *Angelica sinensis* (Oliv.) Diels [Apiaceae; Angelica sinensis R.] (10)	NR	NR
Biqufeng drink (BGF, Decoction, 10)	The Affiliated Wenzhou Hospital of TCM of Zhejiang Chinese Medical University	Partially reported^b^	*Cyathula officinalis* K.C.Kuan [Amaranthaceae; Cyathulae R.] (12), *Dioscorea collettii* var. *Hypoglauca* (Palib.) S.J.Pei & C.T.Ting [Dioscoreaceae; Tokoro Rh.] (35), *Paeonia lactiflora* Pall. [Paeoniaceae; Paeoniae R. Rubra] (12), *Smilax glabra* Roxb. [Smilacaceae; Smilacis Rh.] (20), *Vigna umbellata* (Thunb.) Ohwi & H.Ohashi [Fabaceae; Phaseoli Angularis S.] (12), *Coix lacryma-jobi* var. *ma-yuen* (Rom.Caill.) Stapf [Poaceae; Coicis S.] (20), Body of *Pericaeta communisma* Gate et Hatai [Lumbricidae; Lumbricus] (15), *Lonicera japonica* Thunb. [Caprifoliaceae; Lonicerae Caulis] (20), *Taraxacum mongolicum* Hand.-Mazz. [Asteraceae; Taraxaci H.] (15), *Plantago asiatica* L. [Plantaginaceae; Plantaginis H.] (15)	NR	NR
Qianggu capsule (QG, Capsule, NR)	Beijing Qihuang pharmaceutical	NR	*NR*	NR	NR
***Herbal Medicines + BPs***					
Jingu capsule (JG, Capsule, 12)	Guangdong Second TCM Hospital	Partially reported^c^	*Rehmannia glutinosa* (Gaertn.) DC. [Orobanchaceae; Rehmanniae R. Preparata] (12), *Angelica sinensis* (Oliv.) Diels [Apiaceae; Angelica sinensis R.] (12), *Achyranthes bidentata* Blume [Amaranthaceae; Achyranthis R.] (10), *Cornus officinalis* Siebold & Zucc. [Cornaceae; Corni F.] (12), *Poria cocos* Wolf [Polyporaceae; Poria Sclerotium] (12), *Dipsacus asper* Wall. ex DC. [Caprifoliaceae; Dipsaci R.] (12), *Eucommia ulmoides* Oliv. [Eucommiaceae; Eucommiae C.] (10), *Paeonia lactiflora* Pall. [Paeoniaceae; Paeoniae R. Alba] (10), *Citrus × aurantium f. deliciosa* (Ten.) M.Hiroe [Rutaceae; Citri Unshius Pericarpium Immaturus] (5), *Eleutherococcus nodiflorus* (Dunn) S.Y.Hu [Araliaceae; Acanthopanacis C.] (10), Hornes of *Cervus nippon* Temminck [Cervidae; Cervi Cornus Colla] (6), *Pyritum* (6)	NR	NR
Gushukang (GSK, NR, NR)	Shaowu Municipal Hospital	NR	*NR*	NR	NR
Xianlinggubao (XLGB, NR, NR)	Shaowu Municipal Hospital	NR	*NR*	NR	NR
Jintiange (JTG, NR, NR)	Shaowu Municipal Hospital	NR	*NR*	NR	NR

BP: bisphosphonate, C: cortex, DMARDs: disease-modifying anti-rheumatic drugs, F: fructus, Fl: flos, H: herba, HM: herbal medicine, N: number, NR: not reported, R.: radix.

Ra: ramulus, Rh: rhizoma, S: semen, TCM: traditional Chinese medicine.

*The composition of botanical drugs was the same; however, each dose was different.

^a^ Extraction temperature and time and amount of the provoked extract were reported but not the amount of the initial solvent.

^b^ Only the amount of provoked extract was reported.

^c^ Referred to simply as “boiling”.

In total, 54 botanical drugs were used in the 18 RCTs, including four animal-derived, 49 plant-derived, and one mineral-derived compositions. *Angelica sinensis* (Oliv.) Diels [Apiaceae; Angelica Sinensis Radix] was used the most in eight HMs, followed by *Epimedium brevicornu* Maxim. [Berberidaceae; Epimedii Herba] (in seven HMs); *Cibotium barometz* (L.) J.Sm. [Cyatheaceae; Cibotii Rhizoma] (in six HMs); *Gentiana macrophylla* Pall. [Gentianaceae; Gentianae Macrophyllae Radix]*, Astragalus mongholicus* Bunge [Fabaceae; Astragali Radix]*,* and *Aconitum carmichaelii* Debeaux [Ranunculaceae; Aconiti Lateralis Radix Preparata] (each in five HMs); *Sinomenium acutum* (Thunb.) Rehder & E.H.Wilson [Menispermaceae; Sinomenii Caulis et Rhizoma], *Saposhnikovia divaricata* (Turcz. ex Ledeb.) Schischk. [Apiaceae; Saposhnikoviae Radix], *Ephedra sinica* Stapf [Ephedraceae; Ephedrae Herba], *Achyranthis Radix,* and *Eucommia ulmoides* Oliv. [Eucommiaceae; Eucommiae Cortex] (each in four HMs); *Rehmannia glutinosa* (Gaertn.) DC. [Orobanchaceae; Rehmanniae Radix Preparata], *Dipsacus asper* Wall. ex DC. [Caprifoliaceae; Dipsaci Radix], *Cullen corylifolium* (L.) Medik. [Fabaceae; Psoraleae Semen], and *Spatholobus suberectus* Dunn [Fabaceae; Spatholobi Caulis] (each in three HMs); and *Cistanche deserticola* Ma [Orobanchaceae; Cistanchis Herba], *Cuscuta chinensis* Lam. [Convolvulaceae; Cuscutae Semen], *Salvia miltiorrhiza* Bunge [Lamiaceae; Salviae Miltiorrhizae Radix], Hornes of *Cervus nippon* Temminck [Cervidae; Cervi Cornus Colla], *Paeonia lactiflora* Pall. [Paeoniaceae; Paeoniae Radix Rubra], and *Cornus officinalis* Siebold & Zucc. [Cornaceae; Corni Fructus] (each in two HMs) (Tables 2, 3).

**TABLE 3:** The list of botanical drugs used frequently in HMs prescribed as interventions in RCTs.

Frequency	Botanical drug
8	*Angelica sinensis* (Oliv.) Diels [Apiaceae; Angelica sinensis R.]
7	*Epimedium brevicornu* Maxim. [Berberidaceae; Epimedii H.]
6	*Cibotium barometz* (L.) J.Sm. [Cyatheaceae; Cibotii Rh.]
5	*Gentiana macrophylla* Pall. [Gentianaceae; Gentianae Macrophyllae R.], *Astragalus mongholicus* Bunge [Fabaceae; Astragali R.], *Aconitum carmichaelii* Debeaux [Ranunculaceae; Aconiti Lateralis R. Preparata]
4	*Sinomenium acutum* (Thunb.) Rehder & E.H.Wilson [Menispermaceae; Sinomenii Caulis et Rh]., *Saposhnikovia divaricata* (Turcz. ex Ledeb.) Schischk. [Apiaceae; Saposhnikoviae R.], *Ephedra sinica* Stapf [Ephedraceae; Ephedrae H.], *Achyranthes bidentata* Blume [Amaranthaceae; Achyranthis R.], *Eucommia ulmoides* Oliv. [Eucommiaceae; Eucommiae C.]
3	*Rehmannia glutinosa* (Gaertn.) DC. [Orobanchaceae; Rehmanniae R. Preparata], *Dipsacus asper* Wall. ex DC. [Caprifoliaceae; Dipsaci R.], *Cullen corylifolium* (L.) Medik. [Fabaceae; Psoraleae S.], *Spatholobus suberectus* Dunn [Fabaceae; Spatholobi Caulis]
2	*Cistanche deserticola* Ma [Orobanchaceae; Cistanchis H.], *Cuscuta chinensis* Lam. [Convolvulaceae; Cuscutae S.], *Salvia miltiorrhiza* Bunge [Lamiaceae; Salviae Miltiorrhizae R.], Hornes of *Cervus nippon* Temminck [Cervidae; Cervi Cornus Colla], *Paeonia lactiflora* Pall. [Paeoniaceae; Paeoniae R. Rubra], *Cornus officinalis* Siebold & Zucc. [Cornaceae; Corni F.]

C, cortex; F, fructus; H, herba; HM, herbal medicine; R, radix; RCT, randomized controlled trials; Rh, rhizoma; S, semen.

### 3.3 Meta-analysis of changes in BMD

#### 3.3.1 HM plus DMARDs vs. DMARDs alone

**FIGURE 2:**
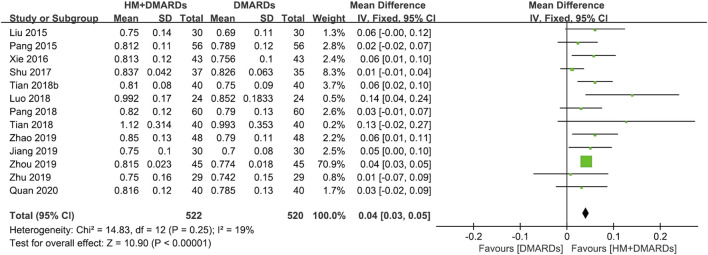
Comparison of BMD at the lumbar spine between HM plus DMARDs vs. DMARDs. CI: confidence interval, DMARDs: disease-modifying anti-rheumatic drugs, HM: herbal medicine, SD: standard deviation.

**FIGURE 3:**
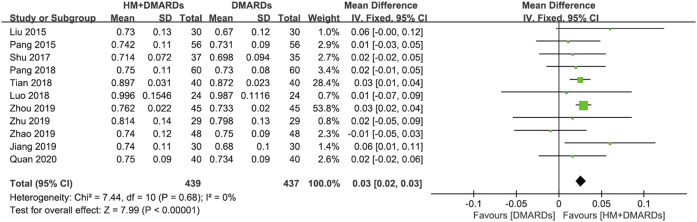
Comparison of BMD at the femoral neck between HM plus DMARDs vs. DMARDs. CI: confidence interval, DMARDs: disease-modifying anti-rheumatic drugs, HM: herbal medicine, SD: standard deviation.

When a meta-analysis was conducted after excluding three studies (Wang, 2018a; Wang and Liu, 2018b; Liu, 2018) that caused heterogeneity, in 13 RCTs that used DMARDs, the HM plus DMARDs group showed a more significant synergistic effect than the DMARDs alone group on the BMD of each site. Compared to that in the DMARDs alone group, BMD improved by 0.04 g/cm^2^ (95% CI: 0.03–0.05; *p* < 0.00001; I^2^ = 19%) in the lumbar spine and 0.03 g/cm^2^ (95% CI: 0.02–0.03; *p* < 0.00001; I^2^ = 0%) in the femoral neck (Figures 2, 3). When treated in parallel with DMARDs and HM, BMD increased by 0.08 g/cm^2^ (95% CI: 0.05–0.12; *p* < 0.00001; I^2^ = 80%) in the lumbar spine and 0.06 g/cm^2^ (95% CI: 0.03–0.08; *p* < 0.00001; I^2^ = 78%) in the femoral neck compared to the corresponding values before treatment (Supplementary Figure S3).

#### 3.3.2 HM plus BPs vs. BPs alone

**FIGURE 4:**
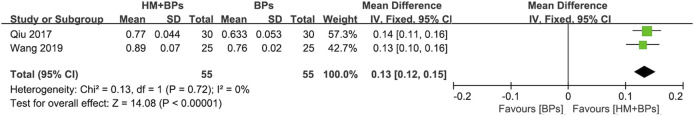
Comparison of BMD at the lumbar spine between HM plus BPs vs. BPs. BPs: bisphosphonates, CI: confidence interval, HM: herbal medicine, SD: standard deviation.

**FIGURE 5:**
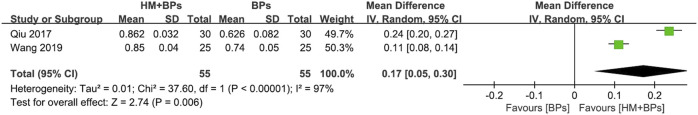
Comparison of BMD at the femoral neck between HM plus BPs vs. BPs. BPs: bisphosphonates, CI: confidence interval, HM: herbal medicine, SD: standard deviation.

In the two RCTs that used BPs, the BMD of both the lumbar spine and femoral neck increased significantly in the HM plus BPs group compared to that in BPs alone group. In each analysis according to the measurement site, a significant difference in BMD improvement was observed compared to the control group in both sites, with 0.13 g/cm^2^ (95% CI: 0.12–0.15; *p* < 0.00001; I^2^ = 0%) in the lumbar spine and 0.17 g/cm^2^ (95% CI: 0.05–0.30; *p* = 0.006; I^2^ = 97%) in the femoral neck (Figures 4, 5). When HM was used in parallel with BPs in the treatment, BMD increased by 0.19 g/cm^2^ (95% CI: 0.17–0.20; *p* < 0.00001; I^2^ = 0%) in the lumbar spine and 0.24 g/cm^2^ (95% CI: 0.20–0.29; *p* < 0.00001; I^2^ = 89%) in the femoral neck compared to before treatment (Supplementary Figure S3).

**FIGURE 6:**
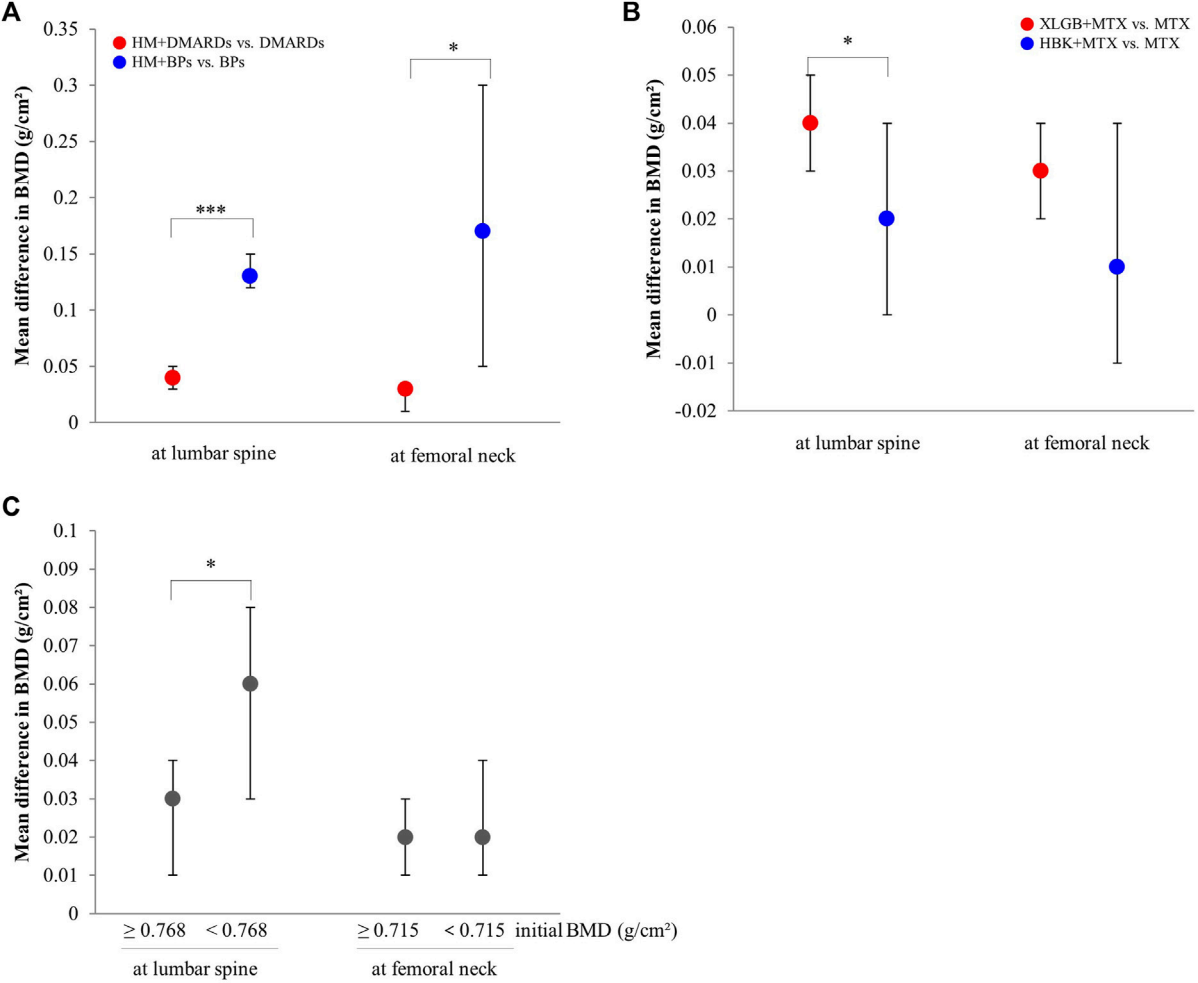
Comparison of the mean difference in BMD by subgroups. **(A)** By the type of combined western medicine. **(B)** By the prescriptions frequently used. **(C)** By the initial BMD scores. BMD: bone mineral density, BPs: bisphosphonates, DMARDs: disease-modifying anti-rheumatic drugs, HBK: Hanbikang-decoction, HM: herbal medicine, MTX: methotrexate, XLGB: Xianlinggubao-capsule. *: The *p*-value of subgroup differences was <0.05. ***: The *p*-value of subgroup differences was <0.0005.

Comparing mean differences in BMD according to the type of co-intervention, the mean difference was significantly greater in both lumbar spine and femoral neck in the HM plus BPs group than in the HM plus DMARDs group (Figure 6A).

#### 3.3.3 Changes in BMD according to frequently prescribed HMs

The frequently used prescriptions in the selected studies were XLGB (Luo et al., 2018; Zhou, 2019; Zhu and He, 2019) and HBK (Pang et al., 2015; Shu, 2017; Quan et al., 2020), which were used in three studies each. All six studies that used XLGB or HBK used MTX as the main Western treatment.

**FIGURE 7:**
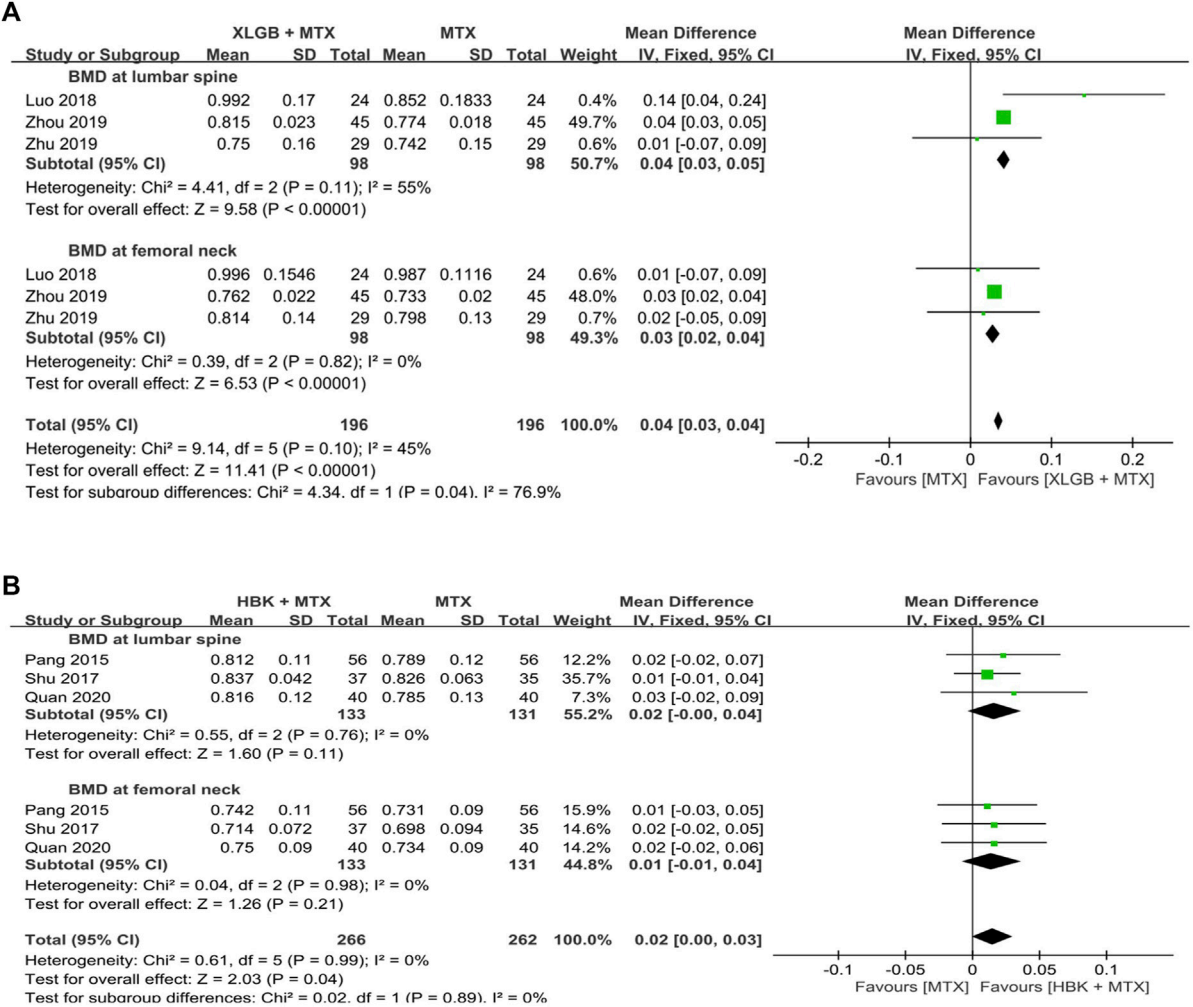
Comparison of BMD between the frequently used HM plus MTX group and the MTX group. **(A)** The XLGB + MTX group vs. the MTX group. **(B)** The HBK + MTX group vs. the MTX group. BMD: bone mineral density, CI: confidence interval, HBK: Hanbikang-decoction, HM: herbal medicine, MTX: methotrexate, SD: standard deviation, XLGB: Xianlinggubao-capsule.

A meta-analysis of BMD after treatment showed that the group treated with XLGB and MTX, or other DMARDs containing MTX, improved BMD by 0.04 g/cm^2^ (95% CI: 0.03–0.05; *p* < 0.00001; I^2^ = 55%) in the lumbar spine and by 0.03 g/cm^2^ (95% CI: 0.02–0.04; *p* < 0.00001; I^2^ = 0%) in the femoral neck. This was higher compared to the DMARDs alone treatment group containing MTX (Figure 7A). At this time, as the heterogeneity was >55%, we conducted a sensitivity analysis, and identified a specific study to be the cause of heterogeneity (Luo et al., 2018). However, the mean difference (MD) and 95% CI was the same before and after excluding the study, therefore, it was retained. In the group treated with HBK and MTX, or other DMARDs, including MTX, BMD was found to be higher after treatment by 0.02 g/cm^2^ (95% CI: −0.00–0.04) in the lumbar spine and by 0.01 g/cm^2^ (95% CI: −0.01–0.04) in the femoral neck, higher than that observed in the DMARDs only treatment group, including MTX (Figure 7B). However, as the *p*-values were *p* = 0.11 in the lumbar spine and *p* = 0.21 in the femoral neck, there was no significant difference from the BMD after treatment of the MTX alone treatment group.

When comparing the two prescriptions, the combined treatment group with XLGB and MTX had a greater BMD difference compared to the control group in the lumbar spine than that observed in the combined treatment group that used HBK and MTX (Figure 6B).

#### 3.3.4 Changes in BMD scores by initial BMD

A meta-analysis was conducted to compare the improvement of BMD between the HM plus WM and WM alone groups. The median value of the initial BMD was measured in the lumbar spine and later in the femoral neck. The group with relatively high and low initial BMD was classified based on the median value. In this analysis, two RCTs that used BPs as intervention (Qiu et al., 2017; Wang, 2019) were excluded because the change in BMD before and after treatment was significantly greater than that observed in studies that used WM as intervention, and both studies involved the group with low initial BMD values, which could excessively affect heterogeneity and results.

In the lumbar spine (median, 0.768 g/cm^2^), the effect of BMD improvement was greater in the HM plus DMARDs subgroup with low initial BMD than in the DMARDs alone group. Conversely, in the femoral neck (median, 0.715 g/cm^2^), there was no significant difference in BMD improvement between the group with relatively high initial BMD value and that with low initial BMD value (Figure 6C).

### 3.4 Changes in bone markers and inflammatory indicators, including BMD

All three studies that used CTX-I as an evaluation index showed a significant reduction effect compared to that in the control group after treatment.

With respect to the bone formation markers, all four studies used BGP as a measurement and reported a significant increase compared to the control group after treatment. Two of the three studies that evaluated ALP showed a significant improvement effect compared to the control group after treatment. However, in one study (Tian et al., 2018a), it was unknown whether there was a significant difference from the control group. All eight studies that evaluated BALP showed significant differences compared to the control group after treatment.

Among the mineral composition and metabolic markers, all studies that used 25-OHD, a urinary Ca/Cr, and PTH as outcome measurements showed significant improvement compared with the control group before and after treatment. Ca increased significantly in seven studies except for one (Tian et al., 2018b) compared to the control group after treatment. However, no significant or unknown effect was observed in two of four studies that used P as evaluation indicators. RANKL, OPG, and RANKL/OPG were used as outcome indices in two of 18 studies and showed significant improvement effects.

**FIGURE 8:**
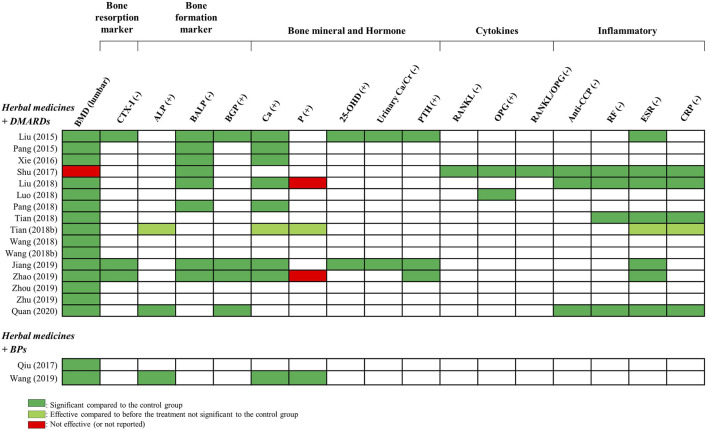
Summary of BMD values and bone marker outcomes in RCTs. 25-OHD: 25-hydroxyvitamin D, ALP: alkaline phosphatase, Anti-CCP: anti-cyclic citullinated peptide, BALP: bone alkaline phosphatase, Ca: calcium, Cr: creatinine, CRP: C-reactive protein, CTX: C-telopeptide of type 1 collagen, ESR: erythrocyte sedimentation rate, OPG: osteoprotegerin, P: phosphorus, PTH: parathyroid hormone, RANKL: receptor activator of nuclear factors κB ligand, RCT, randomized clinical trial, RF, rheumatoid factor.

The results showed that anti-CCP decreased significantly in all three studies, while RF also decreased significantly in all four studies that evaluated these indicators. In addition, all eight studies that evaluated ESR showed a distinct reduction effect compared to the control group, except for one study, in which the effect of the comparison with the control group was unknown. CRP also decreased significantly in all but one of five studies that evaluated it (Figure 8).

### 3.5 Adverse events

Only three of the 18 RCTs mentioned adverse events (Qiu et al., 2017; Shu, 2017; Liu, 2018). Of these, two (Shu, 2017; Liu, 2018) reported side effects, whereas one (Qiu et al., 2017) reported no side effects. The types of adverse events reported were 19 cases of digestive system problems, such as nausea, anorexia, vomiting, mild constipation, and diarrhea; one case of skin problems, such as pruritus; and one case of liver function test abnormality. Nineteen digestive-related symptoms occurred in six cases in the experimental group and in 13 cases in the control group. The most frequently reported adverse event was anorexia. In addition, both skin-related symptoms and liver function test abnormality occurred in the control group (Table 4).

**TABLE 4:** Adverse events reported in each group.

Classification of adverse events	Adverse events	Treatment group	Control group
Digestive system problem (19 cases)	Nausea or vomiting	1	0
	Anorexia	4	9
	Mild constipation	1	0
	Diarrhea	0	2
Skin problem (one case)	Pruritus	0	1
“Not otherwise classified” problem (one case)	LFT abnormality	0	1
Total number of participants who had an adverse event (21 cases)		6	15

LFT, liver function test.

All adverse events shown in both studies were not severe enough to affect the study. Therefore, the study was resumed after confirming that the patients’ condition was improved after symptomatic treatment.

### 3.6 Risk of bias

As there were more than 10 selected studies, we plotted a funnel plot with the *X*-axis set to the effect size and the *Y*-axis set to the standard error. Although there were a few outliers, the left and right sides of the funnel plot were generally symmetrical (Supplementary Figure S4). However, compared to other studies, there were studies with relatively small sample sizes; thus, it is estimated that there is some visual asymmetry.

The risk of bias of each study was assessed and is presented in Supplementary Figures S5, S6. There were two studies reporting the specific randomization process and allocation concealment for participants (Shu, 2017; Liu, 2018), whereas other studies did not mention the randomization method or allocation concealment. None of the selected studies set placebo or sham intervention. There was no risk of missing outcome data because some studies produced dropouts (Shu, 2017; Liu, 2018), whereas the other studies showed no dropouts. In most studies, it was not clear whether participants were blinded for their intervention, which is a concern because it could affect the outcome values of symptom-related indicators. However, four studies (Tian et al., 2018a; Luo et al., 2018; Pang et al., 2018; Wang, 2019) were evaluated as low risk because they used only outcome values that intervention blinding could not affect the results. As all studies did not have a protocol or were not accessible, selection of the reported result was evaluated as some concerns.

### 3.7 Certainty of evidence

All the included studies were RCTs, therefore the study design was evaluated as “high.” Most of the meta-analysis using BMD as an evaluation index was judged as high certainty, whereas some analysis was suspected to have publication bias owing to the asymmetry of the funnel plot. Therefore, the certainty of evidence was “moderate.” The comparison of femoral neck BMD after treatment in the group treated with HM and BPs and only BPs was judged to have very low certainty because of its serious inconsistency and imprecision due to very high heterogeneity (I^2^ = 97%) and very wide 95% CI (0.05–0.30) (Table 5).

**TABLE 5:** GRADE evidence profiles.

Outcome (BMD)	No. of participants (No. Of studies)	Certainty assessment						Absolute effect (95% CI)	Certainty
		Study design	Risk of bias^a^	Inconsistency	Indirectness	Imprecision	Other considerations		
HM + DMARDs *versus* DMARDs alone in the lumbar spine	1,042 (13)	High	Not serious	Not serious	Not serious	Not serious	None	MD 0.04 higher (0.03–0.05 higher)	⊕⊕⊕⊕ High
HM + BPs *versus* BPs alone in the lumbar spine	110 (2)	High	Not serious	Not serious	Not serious	Not serious	None	MD 0.13 higher (0.12–0.15 higher)	⊕⊕⊕⊕ High
HM + DMARDs *versus* DMARDs in the femoral neck	876 (11)	High	Not serious	Not serious	Not serious	Not serious	Publication bias strongly suspected^b^	MD 0.03 higher (0.02–0.05 higher)	⊕⊕⊕○ Moderate
HM + BPs *versus* BPs alone in the femoral neck	110 (2)	High	Not serious	Very serious^c^	Not serious	Serious^d^	None	MD 0.17 higher (0.02–0.30 higher)	⊕○○○ Very low
XLGB + DMARDs *versus* DMARDs alone	196 (3)	High	Not serious	Not serious	Not serious	Not serious	Publication bias strongly suspected^b^	MD 0.04 higher (0.03–0.04 higher)	⊕⊕⊕○ Moderate
HBK + DMARDs *versus* DMARDs alone	264 (3)	High	Not serious	Not serious	Not serious	Not serious	None	MD 0.02 higher (0.00–0.03 higher)	⊕⊕⊕⊕ High

BMD: bone mineral density, BP: bisphosphonate, CI: confidence interval, DMARDs: disease-modifying anti-rheumatic drugs, GRADE: grading of recommendations assessment, development, and evaluation, HBK: Hanbikang-tang, HM: herbal medicine, MD: mean difference, XLGB: Xianlinggubao-capsule.

^a^ All studies were evaluated as “Not serious” because BMD, is an objective indicator; therefore, it is difficult to say that bias would have affected the results.

^b^ Funnel plot is asymmetric.

^c^ High heterogeneity.

^d^ 95% CI, wideness.

## 4 Discussion

Osteoporosis in patients with RA remains a medical challenge that needs to be overcome despite advances in research on the mechanism of RA and drug treatment (Herrera et al., 2015). Several previous studies have reported the efficacy of HMs, including multi-target and multi-compound products, that can promote bone formation, inhibit bone resorption, and reduce ongoing inflammation (Daily et al., 2017; He et al., 2017; Gong et al., 2021; Hong and Lee, 2021; Wang et al., 2021; Han et al., 2022). To evaluate these potential effects of HM as a new alternative treatment for bone loss in patients with RA, a systematic review and a meta-analysis was comprehensively conducted on 15 RCTs. Although 18 RCTs met the eligibility criteria, the heterogeneity was very high when the first meta-analysis was conducted (Supplementary Figures S1, S2), and three studies (Wang, 2018a; Wang and Liu, 2018b; Liu, 2018) were identified to be the cause of increased heterogeneity through sensitivity analysis. Among them, in Liu. (2018) the initial BMD was too high compared to that of other studies, and it is controversial whether the participants were selected well. In the study by Wang. (2018a), the calculated SD was lower than the corresponding value in other studies, which is assumed to have affected heterogeneity. Therefore, a meta-analysis was conducted with 15 RCTs, excluding the three studies that affected heterogeneity. Our data showed that patients with RA having osteoporosis had a higher BMD when administered additional HM compared to using WM alone (DMARDs or BPs). Particularly, when HM was added to DMARDs for approximately 4.2 ± 2.5 months, approximately 9.8% improvement in BMD (improvement by 0.08 g/cm^2^ in the lumbar spine and 0.06 g/cm^2^ in the femoral neck) was observed compared to before treatment, whereas there was an improvement of 0.04 g/cm^2^ in the lumbar spine and 0.03 g/cm^2^ in the femoral neck than when using DMARDs alone (Figures 2, 3). When HM was added to BPs, there was an approximately 34.3% improvement in BMD (approximately 0.19 g/cm^2^ in the lumbar spine and 0.24 g/cm^2^ in the femoral neck) compared to before treatment and an improvement of 0.13 g/cm^2^ in the lumbar spine and 0.15 g/cm^2^ in the femoral neck than when using BPs alone (Figures 4, 5). When HM and WM were combined, the HM plus DMARDs group increased BMD by 0.08 g/cm^2^ in the lumbar spine and by 0.06 g/cm^2^ in the femoral neck than before treatment (Supplementary Figure S3). In general, adult women and men have a peak BMD at the age of 30–39 and 20–29 years, respectively, which gradually decreases with age. On average, men and women in their 50s–60 s reduce BMD by 0.061 g/cm^2^ in the lumbar spine and 0.066 g/cm^2^ in the femoral neck (Zhang et al., 2014). Therefore, the increase in BMD by 0.06 g/cm^2^ by the combination of HM and DMARD for approximately 4.2 months clinically means that bone health improved from 60 to 50 s younger.

In particular, in the HM plus DMARDs group, a lower initial BMD value of the lumbar spine resulted in a greater BMD difference in the DMARDs alone group after treatment (Figure 6C). However, the meta-analysis comparing BMD in the femoral neck after treatment in the HM plus BPs *versus* BPs alone group not only had very high heterogeneity of I^2^ = 97% but also had a “very low” certainty of evidence (Figure 5; Table 5). Therefore, it is controversial to judge the synergetic effect of HM plus BPs on the femoral neck in this study.

This requires caution in interpreting the results, given the low quality of the included RCTs (average Jadad score of 2.5) and the high RoB. In particular, all studies were evaluated as having “some concerns” in RoB 2 (Supplementary Figures S5, S6), because most studies did not mention the specific randomization process, allocation concealment, and blinding using placebo. This can be considered as indicating the low quality of the selected studies; however, the BMD, set as the primary outcome in this study, is an objective indicator in which bias cannot occur by knowing whether the participants took HM. Therefore, it was assumed that it would not have affected the results of the study.

The short duration of treatment (approximately 4.2 months) in the RCTs included in this review and previous studies showing an average increase in BMD of 3.1% when BP was used for 12 months in a population with an average age of 63–78 years (Lyu et al., 2019) may suggest that adding HM to the primary treatment of patients with RA has sufficient potential efficacy for bone loss.

Of the 1,491 participants included in this study, 481 were men and 1,010 women, approximately twice as many women as men. The average age of the study participants was 52.4 ± 7.4 years, and the mean morbidity due to RA was 6.8 ± 1.3 years. Participants’ characteristics were consistent with those of previous studies showing that women had a higher incidence of osteoporosis among patients with RA, especially postmenopausal women (Hauser et al., 2014; Moshayedi et al., 2022). The mean initial BMD of the participants was 0.753 ± 0.109 g/cm^2^ in the lumbar spine and 0.715 ± 0.087 g/cm^2^ in the femoral neck. Compared to an observational study conducted in South China, in which the mean BMD of 405 participants of the same age group with an RA disease duration of 5.5 years was 0.833 ± 0.149 g/cm^2^ in the lumbar spine and 0.667 ± 0.130 g/cm^2^ in the femoral neck (Hu et al., 2021), the initial BMD value of the participants included in our review was found to be lower by 10.6% in the lumbar spine and higher by 6.7% in the femoral neck.

The effect of BMD improvement in the combination treatment group of HM plus WM was also confirmed in the bone markers and inflammation-related indicators identified in each study. Bone resorption markers (CTX-I), bone formation markers (ALP, BALP, and BGP), minor composition and metabolic markers (Ca, P, 25-OHD, urinary Ca/Cr, and PTH), cytokines (RANKL, OPG, and RANKP/OPG), and inflammatory indicators (anti-CCP, RF, ESR, and CRP) were all significantly improved compared to using WM alone in 14 RCTs. In particular, in a meta-analysis of ESR and CRP changes, ESR and CRP decreased significantly by −8.25 mm/h and −3.48 mg/L on average, respectively, compared to the corresponding values in the WM group (Supplementary Figures S7A, B). This was interpreted as the decrease in the inflammatory response being related to the increase in BMD. Evaluating indicators, such as 25-OHD, urinary Ca/Cr, RANKL, OPG, and RANKL/OPG, was considered to have limitations in interpreting positive results because the number of studies using them as evaluation indicators was small, even if each study reported improvement.

Of these, Guizhifuzi-tang was used by Tian et al. (2018a) and presented had the most significant CRP reduction effect. Moreover, *Spatholobus suberectus* Dunn [Fabaceae; Spatholobi Caulis] was the primary botanical drug used in the highest dose. *Spatholobus suberectus* Dunn is known to reduce the influx of inflammatory cells into the vein wall where thrombosis is formed and the serum level of inflammatory cells, inflammatory cytokines, and CRP increase; therefore, it is believed to be related to the results of this study (Tang et al., 2020). *Gentiana macrophylla* Pall. and *E. brevicornu* Maxim. Were the most commonly used botanical drugs in the prescriptions of RCTs that had a reducing effect on ESR. Iridoid, a herbal ingredient of *G. macrophylla* Pall., and Quercetin and Icariin, ingredients of *E. brevicornu* Maxim.*,* are known to have anti-inflammatory effects by inhibiting the expression of cyclooxygenase-2 (COX-2) and inducible nitric oxide synthase (iNOS) in macrophages (Liu et al., 2021; Zhang et al., 2022). Therefore, it is presumed that this mechanism influenced the results of this study.

In most studies, other RA-related symptom indicators, such as pain, swelling, stiffness, and function of the joint, had significant effects compared with those observed in the treatment group before intervention and in the control group after treatment. However, most studies did not specify the measurement method of the symptom index, and even if it was specified, the measurement method used for each study was different; hence, attention should be paid to the interpretation of the results.

The included RCTs used a total of 17 kinds of HMs, and the most frequently used prescriptions were HBK (Pang et al., 2015; Shu, 2017; Quan et al., 2020) and XLGB (Luo et al., 2018; Zhou, 2019; Zhu and He, 2019), used each in three studies. HBK is presumed to alleviate RA activity by reducing the levels of inflammatory cytokines, especially tumor necrosis factor (TNF)-α and interleukin (IL)-1β (Pang et al., 2012). Conversely, XLGB is a commonly used prescription for osteoporosis, as it has already been proven to have anti-osteoporotic effects in previous studies (Hu and Cheng, 2000; Zhu et al., 2012; Wang et al., 2015). In particular, it is believed to exert its anti-osteoporotic effect through processes, such as inhibiting reactive oxygen species, promoting the organonitrogen compound response, and cell migration (Bao et al., 2020). Therefore, our results of using these two prescriptions were consistent with those of previous studies. However, the prescriptions used in the other 12 studies differed in name, composition, and capacity. This can act increase the heterogeneity among studies. However, the heterogeneity was somewhat higher in the meta-analysis comparing XLGB and DMARDs group with the DMARDs alone group. Therefore, we conducted a sensitivity analysis and the study by Luo et al. (2018) was found to be the cause of heterogeneity. However, as the MD and 95% CI before and after excluding the study were same, it was retained. However, all studies did not mention other factors that could affect bone density (e.g., smoking, drinking, exercise, diet, sleep status, etc.). Although it was reported that there was no significant difference in general characteristics between the experimental group and the control group, it was not known whether this meant other factors than age, sex ratio, and duration of RA, so attention should be paid to the interpretation of the results.

The most frequently used botanical drugs in the RCTs were the following six: *A. sinensis* (Oliv.) Diels [Apiaceae; Angelica Sinensis Radix], *E. brevicornu* Maxim. [Berberidaceae; Epimedii Herba], *C. barometz* (L.). J.Sm. [Cyatheaceae; Cibotii Rhizoma], *G. macrophylla* Pall. [Gentianaceae; Gentianae Macrophyllae Radix], *A. mongholicus* Bunge [Fabaceae; Astragali Radix], and *A. carmichaelii* Debeaux [Ranunculaceae; Aconiti Lateralis Radix Preparata] (Table 3). These botanical drugs have been reported to have anti-osteoporotic effects. *Angelica sinensis* (Oliv.) Diels has been reported to have anti-osteoporotic effects by reducing CTX-I and osteocalcin (Lim and Kim, 2014), whereas *C. barometz* (L.) J.Sm. Was reported to have anti-osteoporotic effects by reducing RANKL (Kim et al., 2007). In particular, *G. macrophylla* Pall. and *E. brevicornu* Maxim. Have been reported to have excellent anti-osteoporosis and anti-inflammatory effects. *Gentiana macrophylla* Pall. Inhibits the production of inflammatory cytokines, nitric oxide, and COX-2 (Wang et al., 2013), and reduces the differentiation of RANKL-induced osteoclasts and the expression of Nucleic Factor Kappa B (Yang et al., 2020). Moreover, Icarin, the main ingredient of *E. brevicornu* Maxim., has been reported to reduce COX and inflammatory mediators, such as COX-2 and iNOS, as well as IL-1, IL-6, and TNF-α (Zhang et al., 2022). Therefore, these botanical drugs may be considered for clinical treatment of secondary osteoporosis in patients with RA in the future.

Of the 21 adverse events reported in three of the 18 studies, 90% involved the gastrointestinal system. Of all side effects, 72.4% were found in the control group that only used WM. Therefore, it is worth researching whether adding HMs to WM can attenuate the side effects of DMARDs, including nausea, vomiting, blood problems, muscle pain, and liver problems (Farzaei et al., 2016).

This study had some critical limitations, such as high heterogeneity in some analysis, high bias (i.e., rare blinding and publication), and insufficient reports on the manufacturing process of the HMs used. Especially, concerning HM, all included RCTs did not report the quality control and chemical analysis of HM. Specific manufacturing processes were not specified, and some studies did not mention the dose of components or the component itself. This may mean that the HMs used were not standardized, thus, resulting in heterogeneity of the results. Because of these limitations, caution should be exercised when interpreting the results of this review conclusively. Therefore, based on our findings, further RCTs with low heterogeneity and bias should be conducted in the future aiming at examining the effect of HMs on osteoporosis in patients with RA. Moreover, a description of the extraction process of the HMs and a document on the legal basis for the collection and processing of the HMs used are also necessary. In addition to this problem, all studies should be conducted under complete control of the confounding factors that affect BMD.

Despite these limitations, this study is meaningful in that it is the first systematic review to provide evidence that BMD, inflammation levels, and bone markers are further improved when HM is combined with WM (DMARDs or BP) for treating patients with RA having osteoporosis. In the future, high-quality clinical studies designed to supplement the limitations of this study and existing studies are required.

## Data Availability

The original contributions presented in the study are included in the article/Supplementary Material, further inquiries can be directed to the corresponding author.
