# Supplementary material for: Combination effects of herbal and western medicines on osteoporosis in rheumatoid arthritis: systematic review and meta-analysis
Source: Front Pharmacol. 2023 Aug 10;14:1164898. doi: 10.3389/fphar.2023.1164898 (PMC10448903; doi:10.3389/fphar.2023.1164898)
Supplement: Supplementary file 1 [file DataSheet1.docx]

Supplementary Material

Combination Effects of Herbal and Western Medicines on Osteoporosis in Rheumatoid Arthritis: Systematic Review and Meta-Analysis

**Do Young Kwon, Ji Hyang Gu, Minseok Oh and Eun-Jung Lee***

*** Correspondence:** Eun-Jung Lee: [jungkahn@dju.kr](mailto:jungkahn@dju.kr)

# Supplementary Figures and Tables

## Supplementary Figures


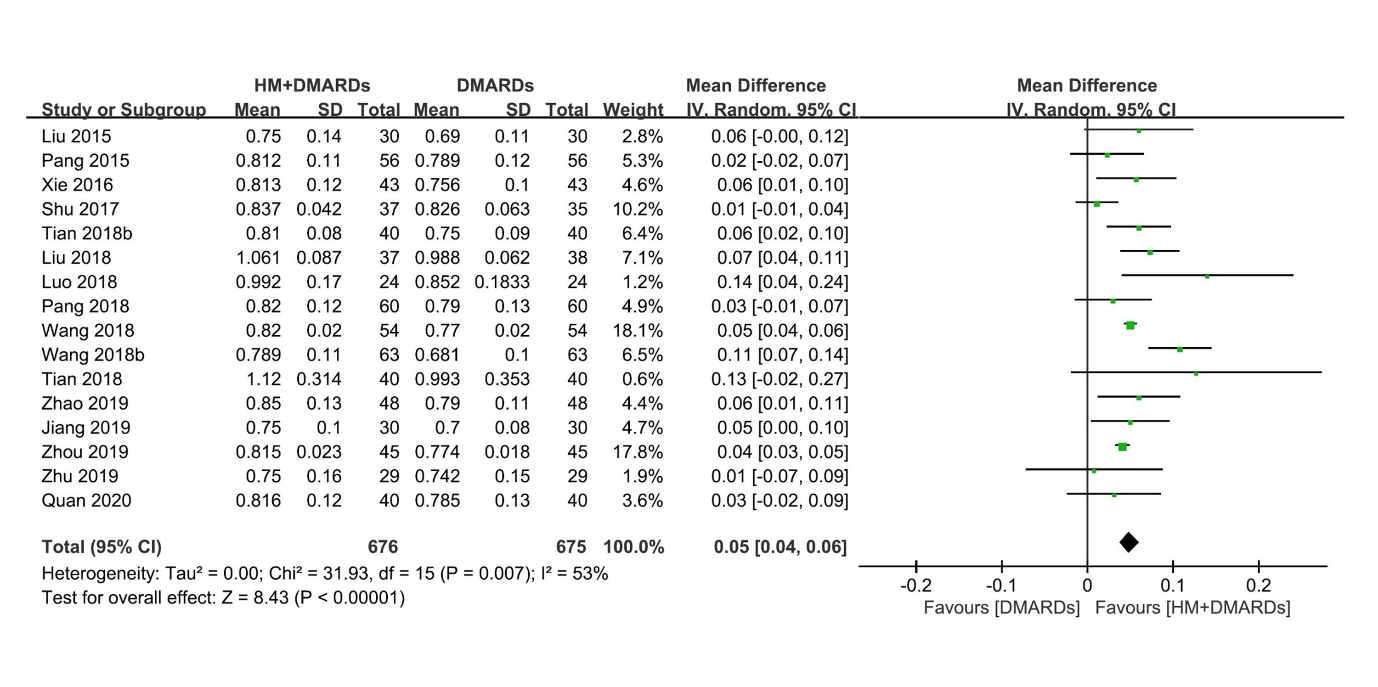


**Supplementary Figure 1.** Comparison of BMD at lumbar spine between HM plus DMARDs vs. DMARDs before exclusion of 3 studies

DMARDs: disease-modifying anti-rheumatic drugs, HM: herbal medicine, SD: standard deviation, CI: confidence interval


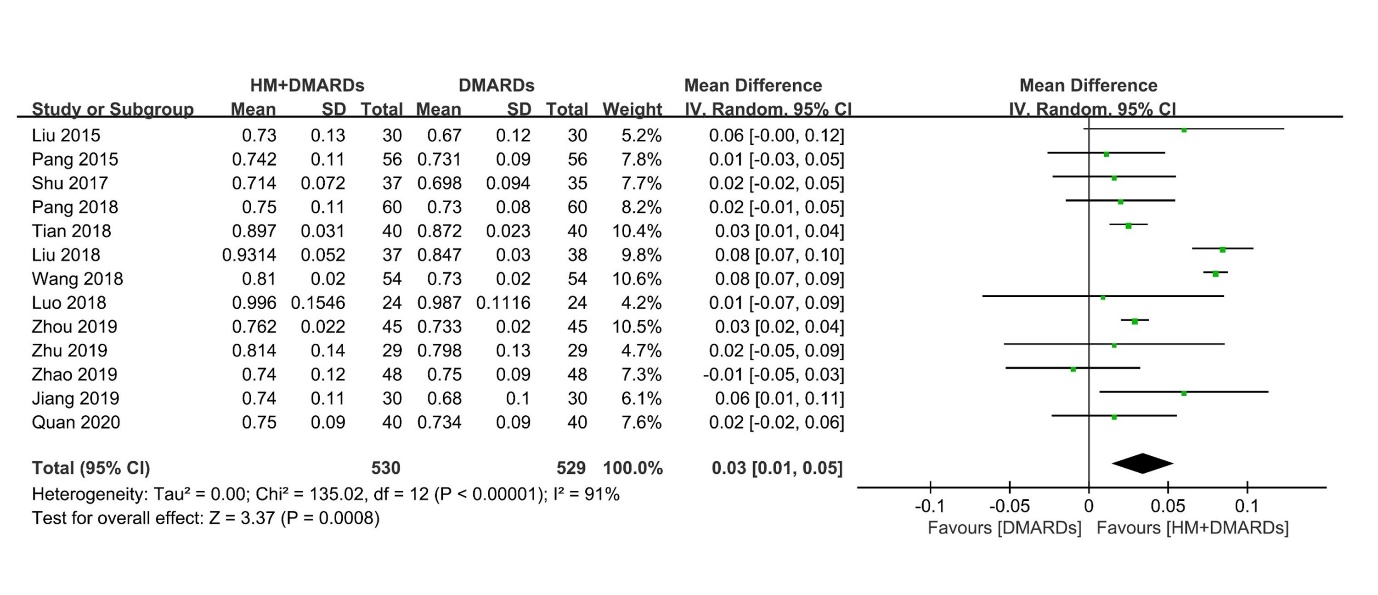


**Supplementary Figure 2.** Comparison of BMD at femoral neck between HM plus DMARDs vs. DMARDs before exclusion of 3 studies

DMARDs: disease-modifying anti-rheumatic drugs, HM: herbal medicine, SD: standard deviation, CI: confidence interval


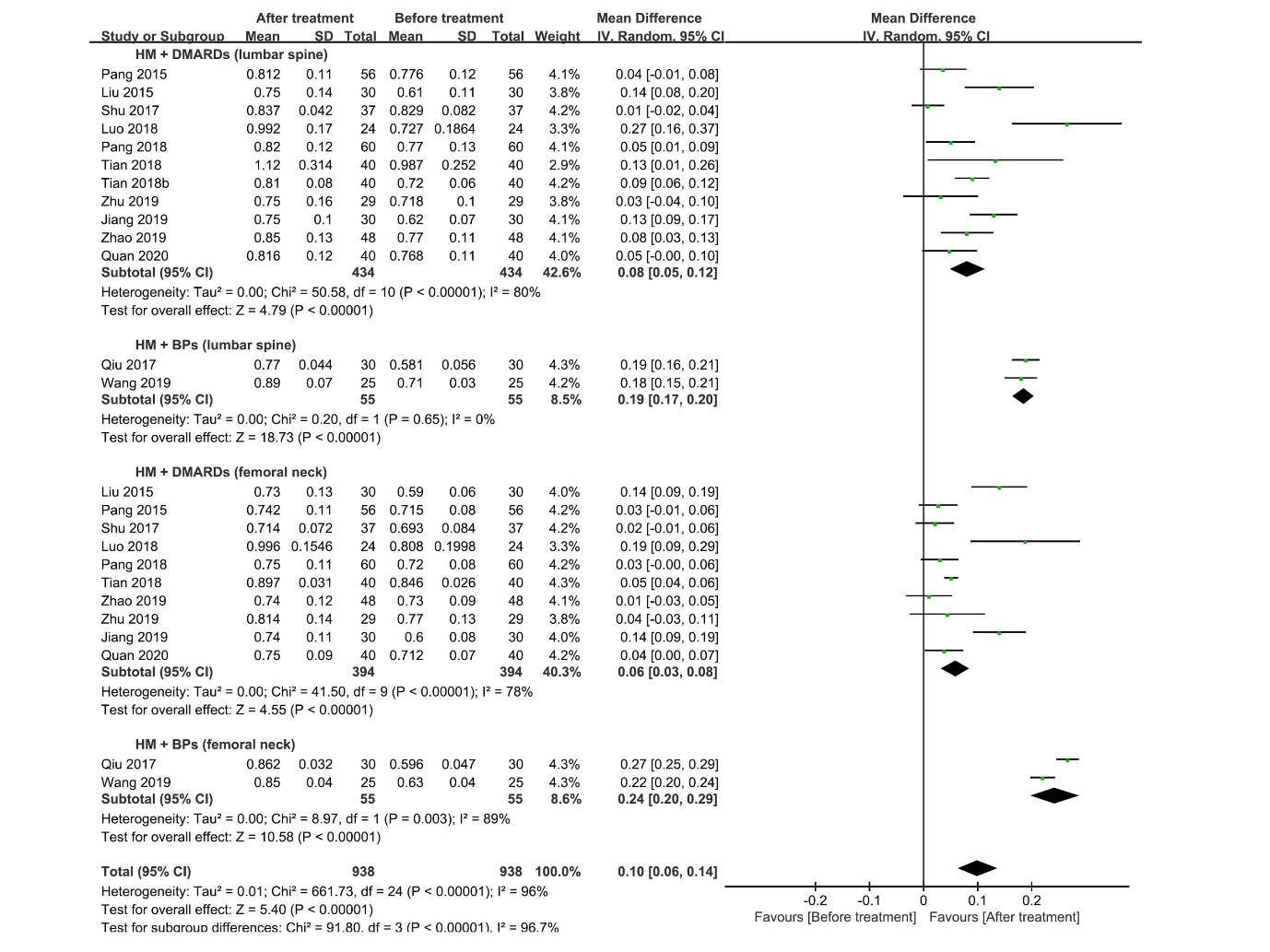


**Supplementary Figure 3.** Change of the BMD score before and after treatment in the HM plus WM group.

BPs: bisphosphonates, DMARDs: disease-modifying anti-rheumatic drugs, HM: herbal medicine, WM: Western medicine, SD: standard deviation, CI: confidence interval


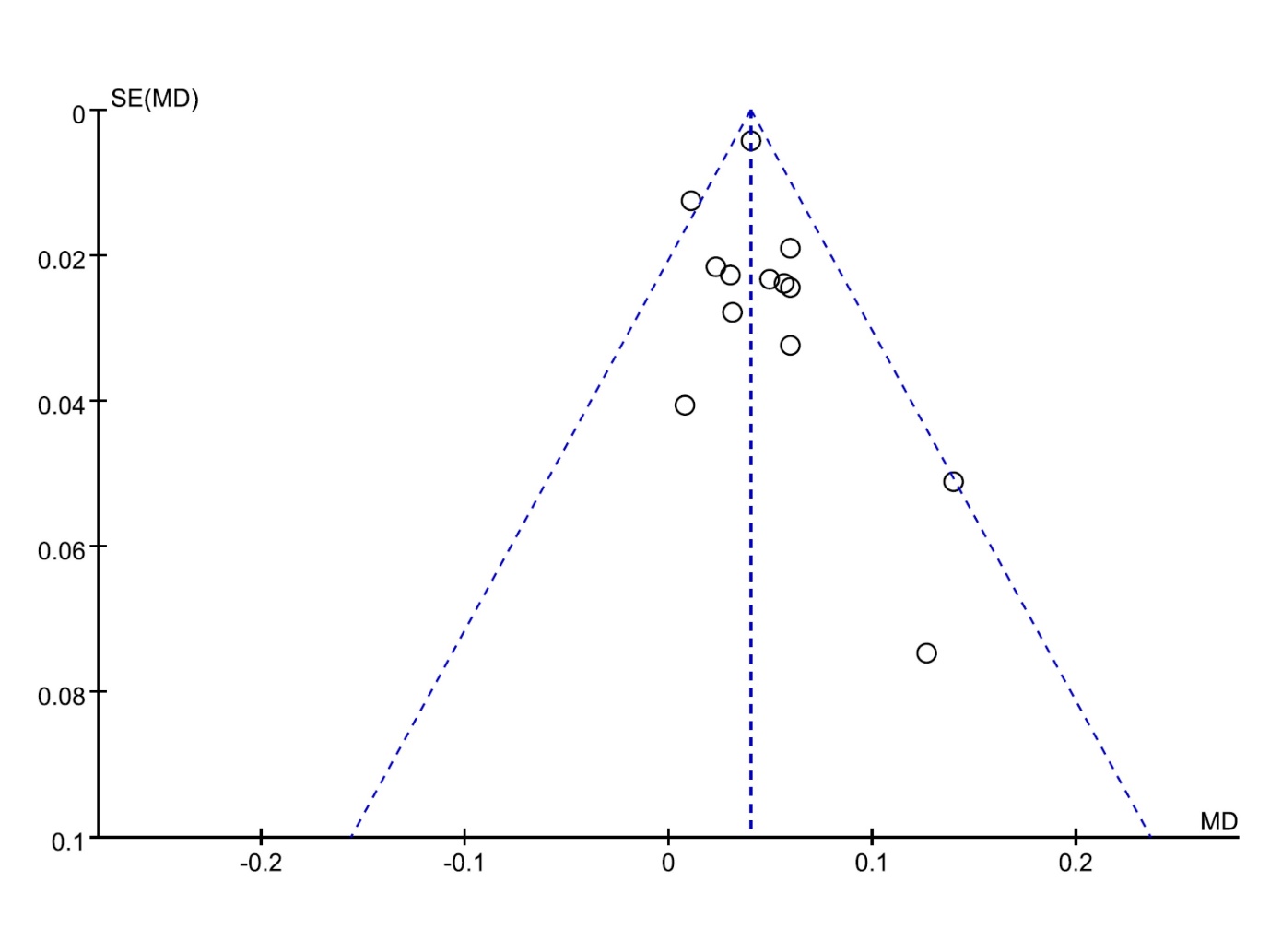


**Supplementary Figure 4**. Funnel plot to evaluate the risk of bias.

MD: mean difference, SE: standard error.


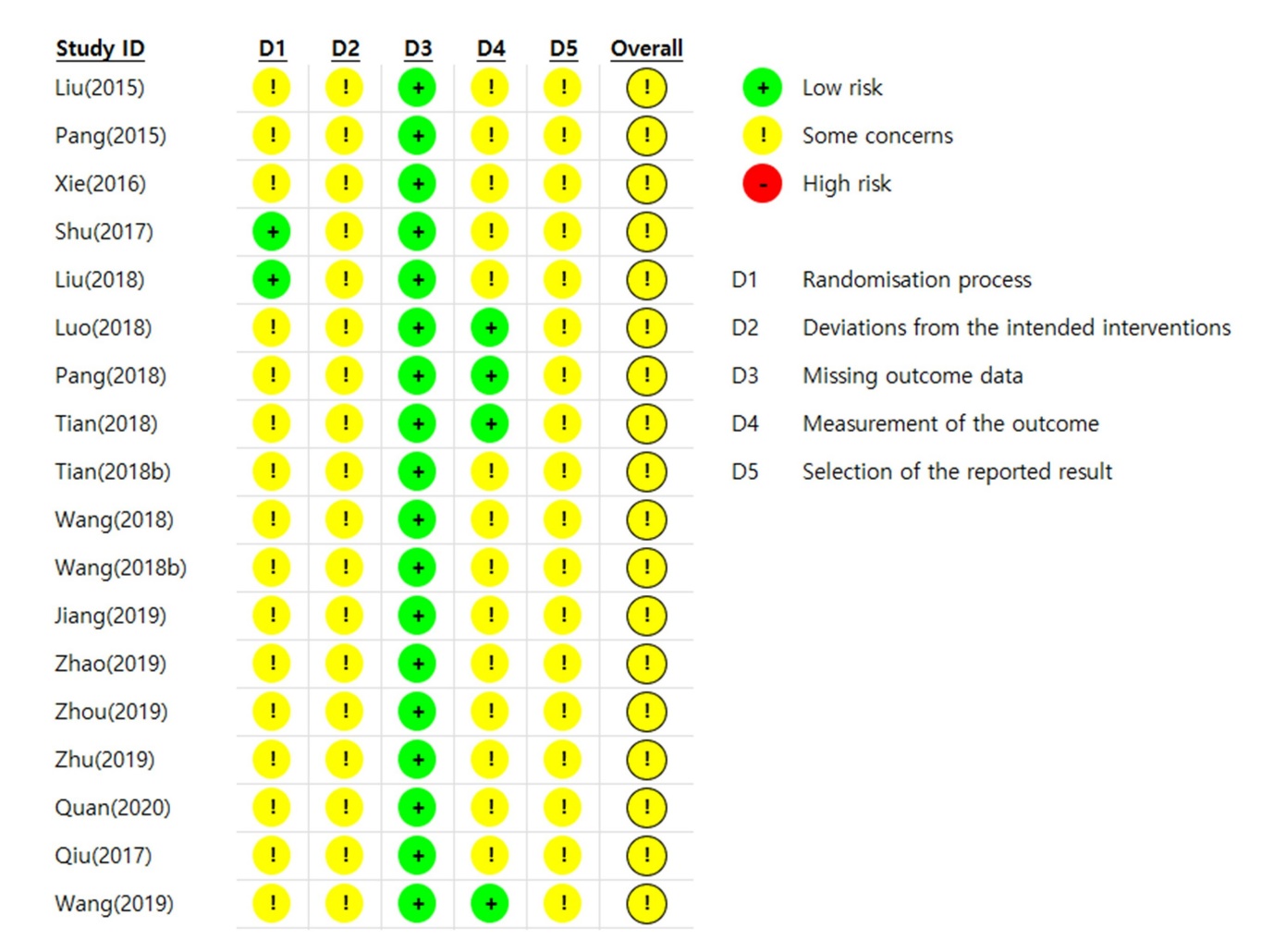


**Supplementary Figure 5**. Assessment of risk of bias.


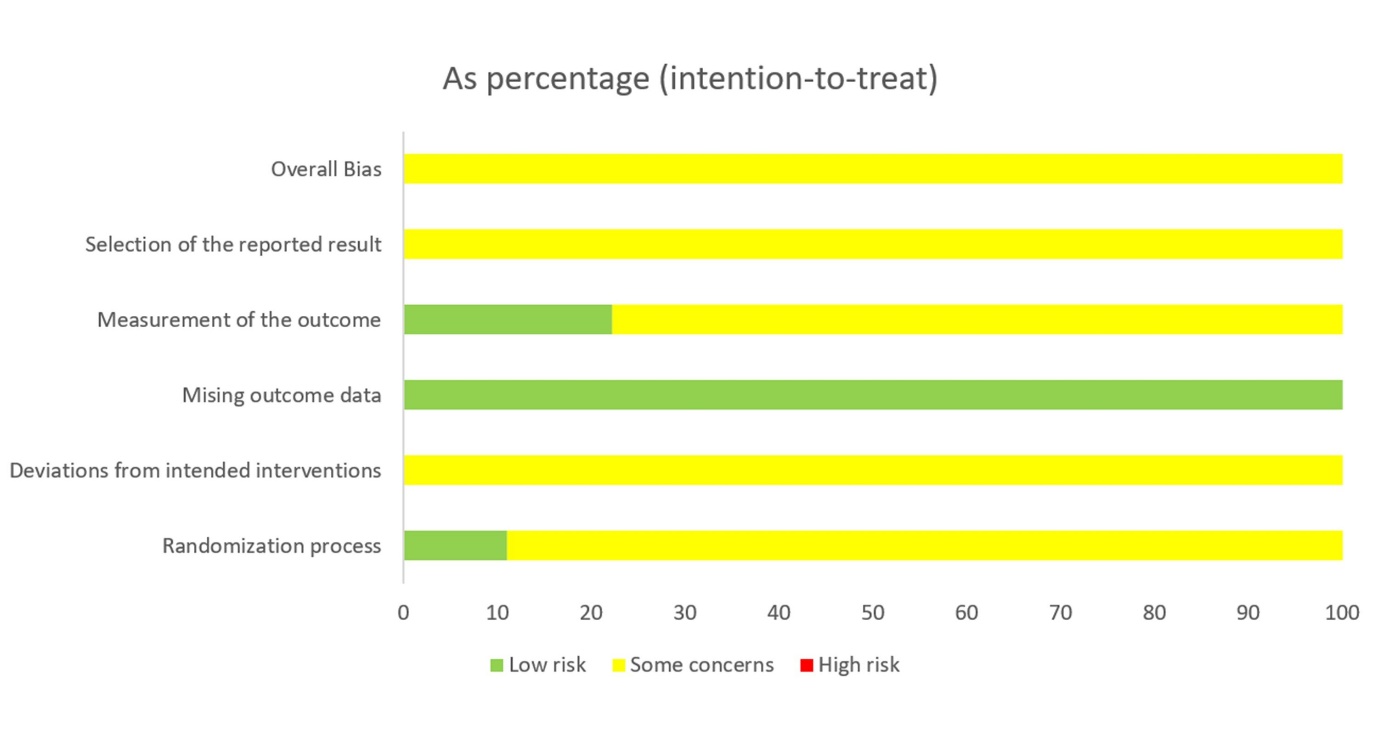


**Supplementary Figure 6**. Risk of bias graph.


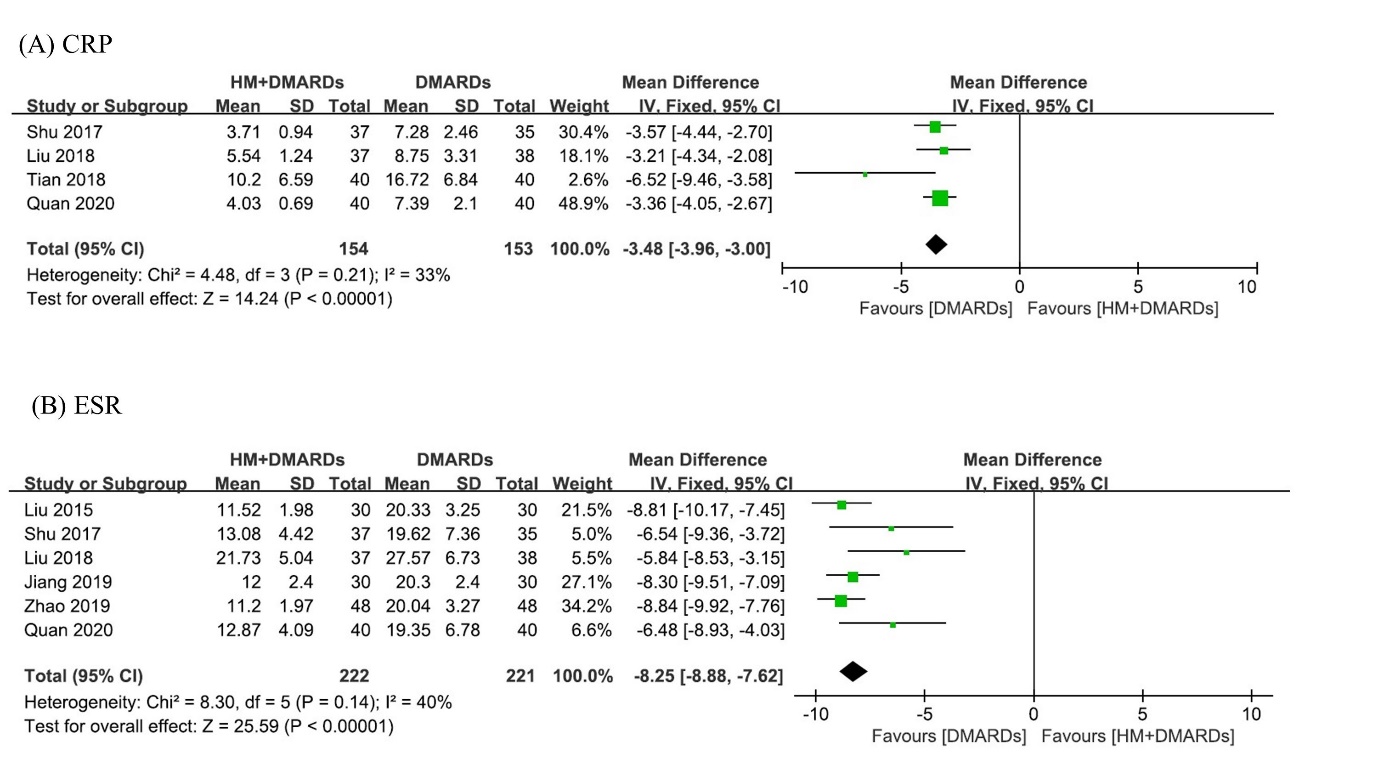


**Supplementary Figure 7**. Comparison of inflammatory indicators between the HM plus WM group and the WM group. (**A**) CRP. (**B**) ESR.

CRP: C-reactive protein, DMARDs: disease-modifying anti-rheumatic drugs, ESR: erythrocyte sedimentation rate, HM: herbal medicine, WM: Western medicine, SD: standard deviation, CI: confidence interval

## Supplementary Tables

**Supplementary Table 1. Search items used in PubMed.**

| No. | Search items |
| --- | --- |
| 1 | Arthritis, Rheumatoid[MeSH Terms] |
| 2 | rheumatic diseases[MeSH Terms] |
| 3 | rheumat*[Title/Abstract] |
| 4 | reumat*[Title/Abstract] |
| 5 | #1 OR #2 OR #3 OR #4 |
| 6 | Osteoporosis[MeSH Terms] |
| 7 | osteoporo*[Title/Abstract] |
| 8 | Bone Diseases, Metabolic[MeSH Terms] |
| 9 | "bone loss"[Title/Abstract] |
| 10 | "low bone densit*"[Title/Abstract] |
| 11 | osteopeni*[Title/Abstract] |
| 12 | #6 OR #7 OR #8 OR #9 OR #10 OR #11 |
| 13 | Medicine, Chinese Traditional[MeSH Terms] |
| 14 | "Chinese medicine*"[Title/Abstract] |
| 15 | Medicine, Korean Traditional[MeSH Terms] |
| 16 | "Korean medicine*"[Title/Abstract] |
| 17 | herb*[Title/Abstract] |
| 18 | TCM[Title/Abstract] |
| 19 | decoction*[Title/Abstract] |
| 20 | "kampo medicine*"[Title/Abstract] |
| 21 | #13 OR #14 OR #15 OR #16 OR #17 OR #18 OR #19 OR #20 |
| 22 | #5 AND #12 AND #21 |

**Supplementary Table 2. Search items used in Embase.**

| No. | Search items |
| --- | --- |
| 1 | ‘rheumatoid arthritis’/exp OR ‘rheumatic disease’/exp OR rheumat*:ab,ti OR reumat*:ab,ti |
| 2 | ‘osteoporosis’/exp OR ‘metabolic bone disease’/exp OR osteoporo*:ab,ti OR ‘bone loss’:ab,ti OR ‘low bone densit*’:ab,ti OR osteopenia*:ab,ti |
| 3 | ‘chinese medicine’/exp OR ‘korean medicine’/exp OR ‘kampo medicine’/exp OR ‘oriental medicine’/exp OR ‘chinese medicine*’:ab,ti OR ‘korean medicine*’:ab,ti OR herb*:ab,ti OR tcm:ab,ti OR decoction*:ab,ti OR ‘kampo medicine*’:ab,ti |
| 4 | #1 AND #2 AND #3 |

**Supplementary Table 3. Search items used in China National Knowledge Infrastructure**

| No. | Search items |
| --- | --- |
| 1 | (AB='风湿') |
| 2 | (AB='骨质疏松'+'骨质减少'+'骨疏松') |
| 3 | (AB='中药'+'中医药'+'提取物'+'汤'+'饮'+'散'+'丸'+'中成药'+'方剂'+'颗粒'+'胶囊'+'口服液') |
| 4 | #1 AND #2 AND #3 |
